# *Pseudomonas aeruginosa* PcrV Enhances the Nitric Oxide-Mediated Tumoricidal Activity of Tumor-Associated Macrophages *via* a TLR4/PI3K/AKT/mTOR-Glycolysis-Nitric Oxide Circuit

**DOI:** 10.3389/fonc.2021.736882

**Published:** 2021-11-25

**Authors:** Hua Yu, Ying Bai, Jing Qiu, Xiaomei He, Junzhi Xiong, Qian Dai, Xingmin Wang, Yuanyuan Li, Halei Sheng, Rong Xin, Lu Jiang, Qiaoqiao Li, Defeng Li, Hong Zhang, Le Zhang, Qian Chen, Jin Peng, Xiaomei Hu, Kebin Zhang

**Affiliations:** ^1^ Clinical Medical Research Center, Xinqiao Hospital, Army Medical University, Chongqing, China; ^2^ Health Management Center, First Affiliated Hospital, Army Medical University, Chongqing, China; ^3^ Administration Department of Nosocomial Infection, Xinqiao Hospital, Army Medical University, Chongqing, China; ^4^ Department of Microbiology, College of Basic Medical Sciences, Army Medical University, Chongqing, China

**Keywords:** *Pseudomonas aeruginosa* PcrV protein, TAM reeducation, tumoricidal efficacy, TLR4/PI3K/AKT/mTOR-glycolysis-NO feedback loop, PcrV–TLR4 interaction

## Abstract

Tumor-associated macrophages (TAMs), which display a tumor-supportive M2 phenotype, are closely related to tumor growth and metastasis. The reprogramming of TAMs toward a tumoricidal M1 profile has emerged as an attractive strategy for cancer immunotherapy. In this study, we found that the intratumoral injection of PcrV protein, a component of the *Pseudomonas aeruginosa* type 3 secretion system, suppressed tumor growth and increased apoptosis, inducible nitric oxide synthase (iNOS) expression, and the percentage of M1-polarized TAMs in tumor tissues. Furthermore, the intratumoral injection of PcrV-primed macrophages exerted a similar tumoricidal effect. *In vitro* analyses revealed that PcrV reeducated TAMs toward an antitumoral M1 phenotype and augmented their nitric oxide (NO)-mediated cytotoxicity against cancer cells. Mechanistically, we found that these effects were dependent on the activation of Toll-like receptor 4 (TLR4)/myeloid differentiation factor 88 (MyD88)-mediated regulation of a PI3K/AKT/mTOR-glycolysis-NO feedback loop *via* direct interaction with TLR4. Collectively, these results revealed a potential role for PcrV in cancer immunotherapy through the targeting of TAM plasticity.

## Introduction

Tumor-associated macrophages (TAMs) form the major component of the immune cell infiltrate in the tumor microenvironment (TME) and are correlated with tumor development and progression. Macrophages infiltrated in the immunosuppressive TME are generally induced into the tumor-supportive M2 phenotype. These M2-like TAMs play roles in supporting tumor growth and metastasis and maintaining an immunosuppressive TME by generating a series of anti-inflammatory and protumoral cytokines and mediators (1). As macrophages are highly heterogeneous and plastic, TAMs exposed to M1-associated stimuli can undergo a reversal from the protumoral M2 to the tumoricidal M1 phenotype. M1-like TAMs exert tumoricidal activity through the production of proinflammatory cytokines (e.g., TNFA and IL12), cytotoxic nitric oxide (NO), and reactive oxygen species (ROS) and the activation of Th1 immune responses *via* increasing the expression of associated major histocompatibility complex class I and class II (MHCI/II) and costimulatory molecules (CD86 and CD80) (2). Accordingly, the reeducation of TAMs from an M2 to an M1 phenotype has emerged as an attractive strategy for cancer immunotherapy.

Recent studies have shown that drugs such as paclitaxel (3) and astragaloside IV (4) can suppress tumor growth by reprogramming TAMs into an M1 phenotype. In addition, several reports have highlighted the potential efficacy of bacteria or their products in antitumoral therapy. For instance, *Mycobacterium bovis*-derived bacillus Calmette–Guérin (BCG) has already been utilized as a preferred first-line treatment for non-muscle invasive bladder carcinoma (5, 6). Additionally, modified lipopolysaccharide (LPS), a Toll-like receptor 4 (TLR4) agonist, has been tested for its antitumoral efficacy in a clinical trial (7). Given that a large number of bacterial components, such as *Brucella abortus* cell-surface protein 31 (BCSP31) protein (8) and *Vibrio cholerae* porin OmpU (9), can induce functional macrophage M1 polarization by activating the TLR-mediated signaling axis (8, 9), these results imply that bacterial products have great potential for exploitation as therapeutic reagents for cancer treatment.

PcrV, a secretory needle tip protein component of the *Pseudomonas aeruginosa* type 3 secretion system (T3SS), helps the translocator proteins PopB and PopD form a functional pore on the target cell membrane (10). PcrV, a V-antigen, elicits protective immune responses against *P. aeruginosa* infection (11, 12). However, the mechanism underlying the PcrV-mediated regulation of the host immune response remains unknown. We have previously reported that PcrV reverses host immune suppression elicited following bacterial biofilm infection *via* the activation of macrophage-mediated immune responses (13). Considering that TAMs exhibit diverse phenotypes and given their function as tumor-resident macrophages, using PcrV to reeducate TAMs and thereby enhance M1 TAM-mediated antitumoral properties is an attractive possibility that requires further investigation.

In the current study, we found that PcrV inhibited tumor growth by reprogramming TAMs to a tumoricidal M1 phenotype. We further found that PcrV-mediated TAM reprogramming potentiated their NO-mediated cytotoxicity against cancer cells, effects that were exerted through the activation of a PI3K/AKT**/**mTOR-glycolysis-NO feedback loop *via* direct interaction with TLR4. Our findings demonstrated that PcrV exerts an immunomodulatory effect on TAMs, providing the basis for further investigation into the potential of PcrV as a therapeutic agent for cancer immunotherapy.

## Materials and Methods

### Cell Culture

Macrophages, mouse Lewis lung cancer (LLC) cells, and HEK293T cells were cultured in Dulbecco’s modified Eagle’s medium (DMEM) (Thermo Fisher, USA) supplemented with 10% fetal bovine serum (FBS) (HyClone, USA) at 37°C with 5% CO_2_.

### Animals and Ethics Statement

Male C57BL/6 mice were purchased from Biocytogen Co., Ltd (Beijing, China). The TLR4^−/−^ and myeloid differentiation factor 88 (MyD88)^−/−^ C57BL/6 mice were provided by Professor Qingwu Yang. Animal experiments were conducted according to the experimental animal guidelines of the Army Medical University of China.

### Expression and Purification of PcrV Protein

Recombinant PcrV protein was expressed from the expression strain *Escherichia coli* JM109/pQE31-PcrV as described previously (13). Protein purification was performed using His-Trap HP affinity columns (GE Healthcare, Sweden), and endotoxin was removed using Detoxi-Gel endotoxin removing gel (Thermo Fisher, USA), according to the manufacturer’s instructions.

### Induction of Bone Marrow-Derived Macrophages and Tumor-Associated Macrophages *In Vitro*

Mouse bone marrow cells were isolated from the tibia and femur of C57BL/6 mice. Bone marrow-derived macrophages (BMDMs) were induced by the administration of mouse macrophage colony-stimulating factor (M-CSF; 30 ng/ml) in DMEM supplemented with 10% FBS and 100 U/ml of penicillin for 7 days at 37°C with 5% CO_2_. For *in vitro* TAM induction, BMDMs were cultured in DMEM/FBS (10%) containing 20% (*v*/*v*) LLC cell culture supernatant for 24 h (14).

### Mouse Tumor Models and Treatment

For PcrV administration, C57BL/6 mice were subcutaneously inoculated with 1 × 10^6^ LLC cells. On day 9, the animals were randomly divided into two groups and were intratumorally injected with either phosphate-buffered saline (PBS; control group) or PcrV (0.5 mg/kg, every 4 days). For the BMDM inoculation experiment, mice were subcutaneously inoculated with 1 × 10^6^ LLC cells (day 0). On days 0 and 7, the animals were peritoneally injected with 200 μl of clodronate liposomes (Liposoma BV, Netherlands) to deplete endogenous macrophages. PBS or PcrV (0.5 mg/kg) was intratumorally injected at days 12, 14, 16, and 18, while the wild type (WT), TLR4^−/−^, or MyD88^−/−^ BMDMs (2 × 10^6^ cells) primed or not with PcrV were intratumorally inoculated at days 16 and 18. At the end of the experiment, the mice were euthanized with pentobarbital, and tumor tissues were harvested for subsequent analyses.

### Fluorescence-Activated Cell Sorting

Tumor tissues were minced and incubated with collagenase type II (1 mg/ml, Thermo Fisher) at 37°C for 40 min to obtain a single-cell suspension. For the staining of extracellular target proteins, 1 × 10^6^ cells were first incubated in a mixture of PBS, 1% FBS, and anti-CD16/32 antibody (BioLegend, #101301) to block non-specific binding and then labeled with the indicated antibodies at room temperature for 30 min. Fluorescence-activated cell sorting (FACS) was performed using a Beckman Coulter Gallios flow cytometer (USA), and the results were analyzed using FlowJo software version 10.0.7 (TreeStar). The following fluorochrome-coupled antibodies were used in the experiment: anti-CD45 (#103115), anti-CD206 (#141711), anti-CD3 (#100235), anti-CD4 (#100203), anti-CD8 (#100733), anti-NK1.1 (#108709), and anti-Gr1 (#108425) (all from BioLegend); and anti-CD11b (#12-0112), anti-F4/80 (#11-4801), anti-CD11c (25–0114), anti-MHCII (#17-5321), and anti-CD86 (#17-0862) (all from eBioscience) antibodies; the fixable viability dye eFluor 506 was from eBioscience (#65-0866).

### TUNEL Assay

Tissue apoptosis was detected using the TdT-mediated dUTP nick end labeling (TUNEL) assay by staining the tissue sections using an *In Situ* Cell Death Detection Kit, POD (Roche, USA), at 37°C for 30 min. Nuclei were counterstained with Hoechst 33342, and images were obtained by laser scanning confocal microscopy (Leica TCS SP5, Germany).

### Immunofluorescence Staining

Murine macrophages (Raw264.7 cells) cultured on coverslips were treated with PcrV (10 μg/ml) for 24 h, fixed in 4% paraformaldehyde at room temperature for 15 min, and stained with antibodies targeting TLR4 (ProteinTech, #19811-1-AP) and PcrV (generated in our laboratory). For tissue staining, paraffin-embedded tumor tissues were sectioned and stained first with antibodies against inducible NO synthase (iNOS) (ProteinTech, # 18985-1-AP), ARG1 (ProteinTech, # 16001-1-AP), and/or F4/80 (Abcam, #ab6640) and then with the corresponding secondary antibodies (labeled with fluorescein isothiocyanate (FITC) or Alexa Fluor 647). Nuclei were counterstained with 4′,6-diamidino-2-phenylindole (DAPI) (Beyotime, China). Images were acquired by laser scanning confocal microscopy (Leica).

### Co-culture and Detection of Apoptosis

BMDMs were seeded at 5 × 10^5^ cells/well in a six-well plate and treated or not with PcrV (10 μg/ml) for 24 h. After the supernatants were discarded, the macrophages (lower chamber of a Transwell plate [0.4-μm pore; Corning, USA]) were co-cultured with LLC cells (upper chamber) in DMEM/FBS (10%) at a ratio of 1:1 for another 24 h. LLC cells were harvested and stained using an Annexin V/7-AAD apoptosis detection kit (BD Biosciences, USA) according to the manufacturer’s instructions. Apoptosis was determined by FACS.

### Western Blotting

Protein extraction and concentration determination were performed following the manufacturer’s instructions (Beyotime). Primary antibodies targeting iNOS (BioLegend, #696802), p-AKT (Ser473) (CST, #4060), AKT (Epitomics, #1081), p-mTOR (Ser2448) (CST, #5536), mTOR (CST, #2983), COX2 (ProteinTech, #12375-1-AP), vimentin (CST, #5741), HA tag (CST, #3724), His tag (ProteinTech, #10001-0-AP), and GAPDH (ProteinTech, #60004-1-Ig) were used in this study. The relative optical densities of the protein bands were quantified using ImageJ software.

### RNA Extraction and Real-Time Quantitative PCR

Total RNA extraction, reverse transcription, and RT-qPCR were performed according to the manufacturer’s protocols. Primers were provided in the Supplementary Material (Table S1).

### Cytokine Level

The levels of TNFA and IL12 p40/70 in the cell culture supernatants were quantified by ELISA kits (BD Biosciences, USA) according to the manufacturer’s instructions.

### Detection of Nitric Oxide Level

NO level in the culture supernatants was measured by Griess reagent (Beyotime, China) according to the manufacturer’s instruction.

### Measurement of Intracellular Reactive Oxygen Species

Cells were incubated with 2′7′-dichlorodihydrofluorescein diacetate (H2DCFDA) (Santa Cruz, USA) dye in DMEM at a final concentration of 5 μM at room temperature for 30 min. Intracellular ROS was measured by FACS.

### Analysis of Lactic Acid Level

Lactic acid level in cell supernatant was measured according to the manufacturer’s instruction (Dojindo Laboratories, Japan).

### Extracellular Acidification Rate Determination

Cells were cultured in a Seahorse XFp cell culture microplate (Agilent, USA) at 5 × 10^4^ cells/well. After pretreatment with the corresponding inhibitors or PcrV, the cells were washed with Seahorse XF Base Medium (103193-100, Agilent) supplemented with 2 mM of glutamine (103579, Agilent). The extracellular acidification rate (ECAR) was measured using a Seahorse XFp Glycolysis Stress Test Kit (103020-100, Agilent) with a Seahorse XFp Analyzer (Agilent) according to the manufacturer’s instructions.

### Migration Assay

LLC cells (3 × 10^4^ cells in serum-free DMEM) that had been serum-starved overnight were seeded in the upper chamber of a Transwell insert (8-μm pore, 24-well, Corning). DMEM supplemented with 20% FBS and with or without PcrV (20 μg/ml) was added to the lower chamber as a chemoattractant. After incubation for 24 or 48 h, the non-migrated LLC cells in the upper chambers were removed. Migrated cells in the bottom chamber were fixed and then stained with 0.5% crystal violet solution for 5 min. The number of migrated cells was counted under an optical microscope (Olympus, Tokyo, Japan).

### Cytotoxicity Assay

Tumor cells and TAMs were treated with various concentrations of PcrV for 48 h. Cytotoxicity was assessed using a Cell Counting Kit-8 (CCK-8; Dojindo Laboratories, Japan) according to the manufacturer’s protocol.

### ATP Assay

Cells were lysed in lysis buffer, and the ATP concentration was tested using an enhanced ATP assay kit (Beyotime) according to the manufacturer’s protocol. The luminescence data as determined using a microplate reader (Varioskan Flash, Thermo Fisher) were normalized against protein concentration.

### Co-Immunoprecipitation

HEK293T cells were transfected with the pCDNA3.1-TLR4 vector using Lipofectamine 2000 (Invitrogen, USA) and cultured in an incubator at 37°C with 5% CO_2_ for 48 h. The obtained cell lysates were incubated with purified PcrV (5 μg) at 4°C for 4 h before the addition of 20 μl of His Mag Sepharose™ Ni (GE Healthcare) and incubation at 4°C for 1 h. PcrV and TLR4 were detected by Western blotting using anti-His and anti-HA-tag antibodies, respectively.

### Surface Plasmon Resonance

For surface plasmon resonance (SPR) analysis, 30 μg/ml of human recombinant TLR4 protein containing a 6×His tag (Abcam, #ab233665) was fixed on the NH2 sensor chip (Nicoya, Canada) using Amine Coupling Kit (Nicoya). Then, PcrV at the indicated concentrations was sequentially injected into the chamber in PBS running buffer. TLR4-PcrV interaction was detected using OpenSPR (Nicoya). The parameters of the binding reactions were calculated and analyzed using Trace Drawer software (Nicoya).

### Statistical Analysis

Data were analyzed using unpaired Student’s *t*-tests or one-/two-way ANOVA in GraphPad Prism version 7.0. Tumor volume data were reported as means ± SEM. Other data were expressed as means ± SD.

## Results

### PcrV Inhibits Tumor Growth by Reprogramming Tumor-Associated Macrophages to a Tumoricidal M1 Phenotype

**Figure 1:**
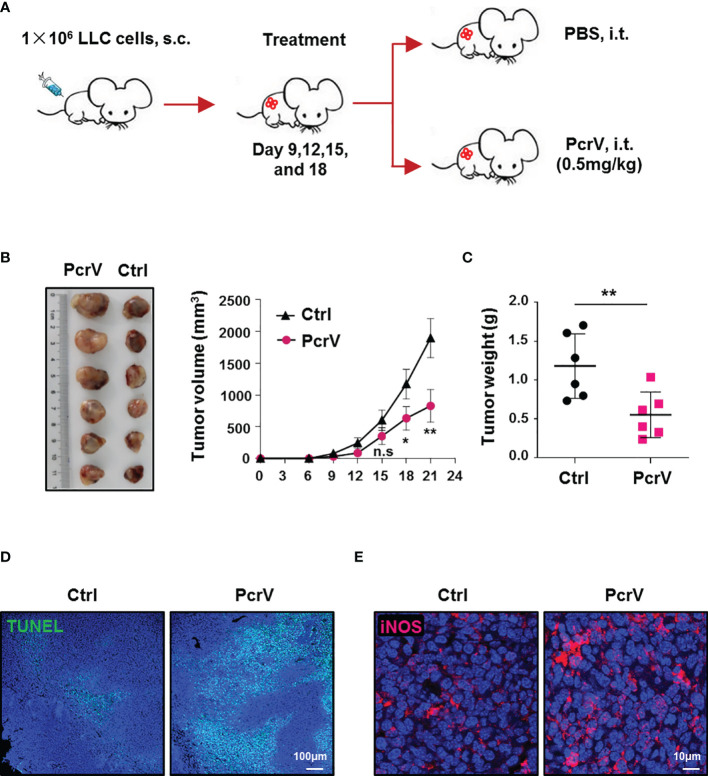
PcrV-mediated suppression of tumor growth is associated with the increased levels of apoptosis and iNOS expression in tumor tissues. Mice bearing LLC cell-derived tumors were treated with PBS or PcrV. **(A)** Schematic of mouse models used. Tumor growth **(B)** and weight **(C)** were measured in LLC tumor-bearing mice treated with PBS or PcrV. Apoptosis **(D)** and iNOS protein level **(E)** in tumor tissues were detected by TUNEL assay and immunofluorescence staining, respectively. Data were expressed as means ± SEM (**B**, *n* = 6) or means ± SD (C, *n* = 6) and were analyzed by two-way ANOVA **(B)** or unpaired Student’s t-test **(C)**. *p < 0.05; **p < 0.01. n.s, no significance; s.c, subcutaneous injection; i.t., intratumoral injection; iNOS, inducible nitric oxide synthase; LLC, Lewis lung cancer; PBS, phosphate-buffered saline.

As PcrV has been reported to activate the host immune response (11, 12), we investigated whether PcrV exerts similar effects in the immunosuppressive TME. First, we assessed the efficacy of PcrV *in vivo* by subcutaneously inoculating LLC cells into the right flanks of C57BL/6 mice followed by intratumoral injection of PcrV (**Figure 1A**). Compared with the control group, PcrV treatment decreased tumor growth (**Figure 1B**) and weight (**Figure 1C**). We previously demonstrated that PcrV significantly increased NO production in normal BMDMs (13). Considering that NO-mediated cytotoxicity is associated with tissue apoptosis and the inhibition of tumor growth, we then evaluated the levels of tumor cell apoptosis and the expression of the NO-generating enzyme—iNOS—in PcrV-treated tumor tissues. The results showed that PcrV treatment increased the levels of apoptosis (**Figure 1D**) and iNOS expression (**Figure 1E**) in the tumor tissues, indicating that PcrV-induced NO generation is associated with the suppression of tumor growth. In addition, we also treated LLC cells and TAMs with PcrV to assess whether PcrV administration might directly cause cytotoxicity or affect tumor cell growth and metastasis. We found that at the concentrations tested, PcrV did not cause significant cytotoxicity against either tumor cells or TAMs (**Supplementary Figure 1A**). Moreover, PcrV treatment did not affect the expression levels of genes (e.g., *Cox2*, *Mmp9*, *Vegfa*, and *Hif1a*; **Supplementary Figure 1B**) or proteins (e.g., COX2 and vimentin; **Supplementary Figure 1C**) related to tumor growth and metastasis, or the metastatic ability of LLC cells (**Supplementary Figure 1D, E**).

**Figure 2:**
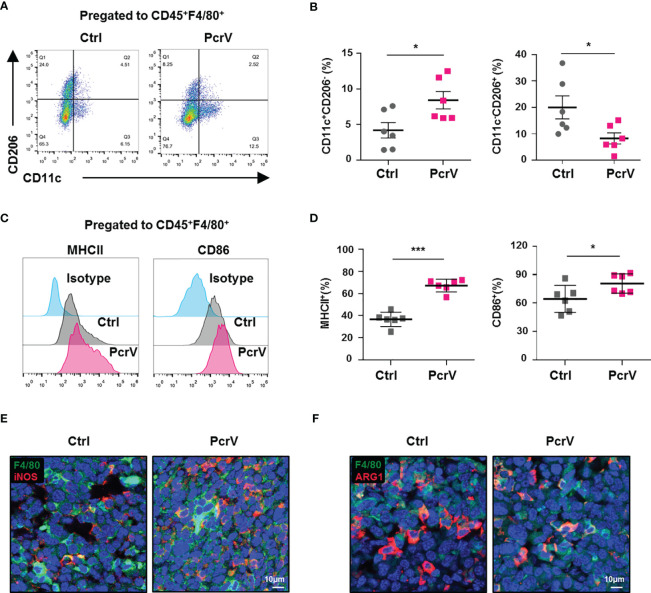
PcrV reeducates tumor-associated macrophages (TAMs) into a tumoricidal M1 phenotype *in vivo*. Mice bearing LLC cell-derived tumors were treated with PBS or PcrV. **(A–D)** FACS analysis of the percentages of M1 (CD45^+^F4/80^+^CD11c^+^CD206^−^, CD45^+^F4/80^+^MHCII^+^, and CD45^+^F4/80^+^CD86^+^) and M2 (CD45^+^F4/80^+^CD11c^−^CD206^+^) TAMs in tumor tissues. **(E, F)** Immunofluorescence staining for visualizing F4/80^+^iNOS^+^ M1 and F4/80^+^Arg1^+^ M2 TAMs in tumor tissues. Data were expressed as means ± SD [**(B, D)**, *n* = 6] and analyzed by unpaired Student’s *t*-test. *p < 0.05 and ***p < 0.001. Ctrl indicates control. LLC, Lewis lung cancer; PBS, phosphate-buffered saline; FACS, fluorescence-activated cell sorting.

Given that immune cells infiltrated in the TME, such as TAMs, CD4^+^ and CD8^+^ T cells, NK cells, and myeloid-derived suppressor cells (MDSCs), play a critical role in modulating antitumoral immune responses (15), we further analyzed the ratios of these cells in the TME following PcrV treatment. The results showed that exposure to PcrV did not significantly affect the percentages of CD4^+^ and CD8^+^ T cells, NK cells, or MDSCs in tumor tissues (**Supplementary Figure 2A**–**F**). However, even though PcrV treatment did not alter the percentage of TAMs infiltrated in the TME (**Supplementary Figure 2G, H**), the ratios of M1-polarized TAMs (F4/80^+^CD11c^+^CD206^−^, F4/80^+^MHCII^+^, and F4/80^+^CD86^+^ macrophages) were increased in PcrV-treated tumor tissues (**Figures 2A**–**D**), whereas those of M2-polarized TAMs (F4/80^+^CD11c^−^CD206^+^ macrophages) were decreased (**Figures 2A, B**). Subsequent analysis of iNOS^+^ TAMs by immunofluorescence (IF) staining indicated that PcrV treatment increased the percentage of F4/80^+^iNOS^+^ M1 TAMs (**Figure 2E**), whereas that of F4/80^+^ARG1^+^ M2 TAMs was decreased (**Figure 2F**). Collectively, these results suggested that PcrV reeducates TAMs to a tumoricidal M1 phenotype *in vivo*.

To determine whether PcrV directly affects TAM polarization *in vitro*, we generated *in vitro*-trained TAMs by incubating BMDMs with LLC cell culture supernatant. These cells showed increased expression of genes related to tumor growth (*Cox2* and *Vegfa*), metastasis (*Cox2*, *Mmp2*, and *Mmp9*), and immune suppression (*Ido2*) (**Supplementary Figure 3A**), indicating that TAM generation *in vitro* had been successful. The levels of tumoricidal M1 polarization markers, including *iNos*, *Cd11c*, *MhcI*, *MhcII*, *Cd86* (**Supplementary Figure 3B**), iNOS (**Supplementary Figure 3C**), NO (**Supplementary Figure 3D**), IL12 p40/70, TNFA (**Supplementary Figure 3E**), MHCII, and CD86 (**Supplementary Figure 3F, G**), were found to be higher in PcrV-primed than mock-treated TAMs, whereas the levels of the protumoral M2 polarization markers *c-Myc*, *Egr2*, *Fn1*, *Arg1* (13), and *Cd206* were lower (**Supplementary Figure 3B**), demonstrating that PcrV directly reprograms TAMs to an antitumoral M1 profile *in vitro*.

**Figure 3:**
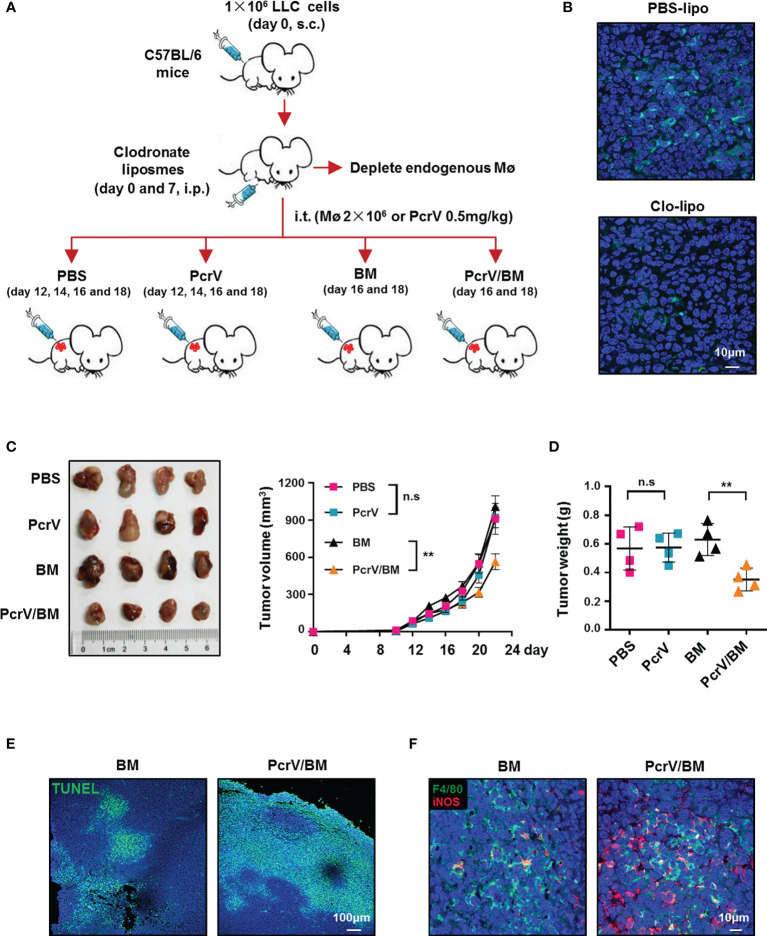
PcrV-primed macrophages suppress tumor growth by increasing the levels of apoptosis and iNOS expression in tumor tissues. **(A)** Schematic of the mouse models used. **(B)** Immunofluorescence staining of F4/80^+^ macrophages in the tumor tissues (harvested on day 16) of mice treated with PBS liposomes or clodronate liposomes (day 0 and 7). Tumor growth **(C)** and weight **(D)** were measured in LLC cell-derived tumor-bearing mice treated with PBS, PcrV, or bone marrow-derived macrophages (BMDMs) primed or not with PcrV. **(E)** Apoptosis was detected by TUNEL assay. **(F)** iNOS protein level and F4/80^+^iNOS^+^ M1 TAMs in tumor tissues were detected by immunofluorescence staining. Data were expressed as means ± SEM (**C**, *n* = 4) or means ± SD (**D**, *n* = 4), and were analyzed by two-way ANOVA **(C)** or unpaired Student’s *t*-test **(D)**. **p < 0.01. s.c, subcutaneous injection; i.p., intraperitoneal injection; i.t., intratumoral injection; BM, BMDMs; PBS-lipo, PBS liposomes; Clo-lipo, clodronate liposomes; iNOS, inducible nitric oxide synthase; PBS, phosphate-buffered saline; LLC, Lewis lung cancer. n.s, no significance.

To further determine whether the PcrV-mediated M1 polarization of TAMs is responsible for tumor growth inhibition, LLC tumor-bearing mice were peritoneally injected with clodronate liposomes to deplete endogenous macrophages, after which the mice were intratumorally injected with PBS, PcrV, or BMDMs primed or not with PcrV (**Figure 3A**). The results showed that clodronate liposome treatment reduced macrophage infiltration in tumor tissues (**Figure 3B**). The treatment with PcrV-primed BMDMs decreased tumor growth (**Figure 3C**) and weight (**Figure 3D**) and increased the levels of apoptosis (**Figure 3E**) and iNOS expression (**Figure 3F**), and the percentage of iNOS^+^F4/80^+^ TAMs (**Figure 3F**) in tumor tissues; however, PcrV treatment did not affect either tumor growth (**Figure 3C**) or weight (**Figure 3D**) in mice depleted of endogenous macrophages due to clodronate liposome administration. Collectively, these results demonstrated that the PcrV-mediated tumoricidal effect is associated with the reprogramming of TAMs to an M1 phenotype.

### PcrV-Primed Tumor-Associated Macrophages Induce the Apoptosis of Cancer Cells by Enhancing Nitric Oxide-Associated Cytotoxicity *In Vitro*

**Figure 4:**
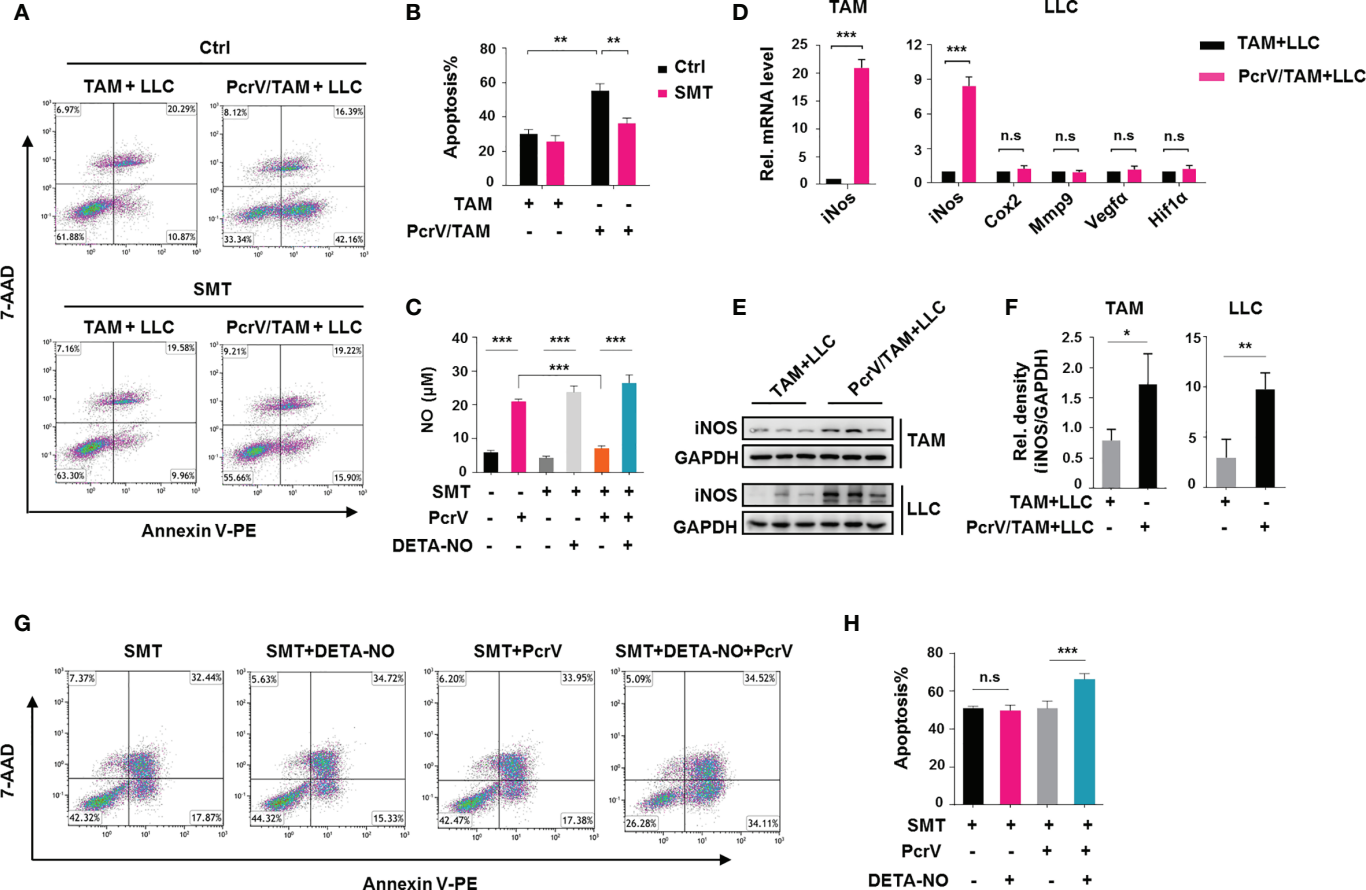
PcrV-primed tumor-associated macrophages (TAMs) induce the apoptosis of tumor cells by enhancing nitric oxide (NO)-associated cytotoxicity *in vitro*. Bone marrow-derived macrophages (BMDMs) were treated or not with PcrV (10 μg/ml) for 24 h and then co-cultured with LLC cells for another 24 h For the inhibition of nitric oxide (NO) production, BMDMs were pretreated with *S*-methyl thiourea (SMT; 0.5 mM) for 2 h followed by exposure to PcrV. To increase the NO level, the culture medium was supplemented with the NO donor DETA-NO (20 μM). **(A, B)** LLC cell apoptosis was detected by FACS using Annexin V/7-AAD staining. **(C)** The NO level in the macrophage-LLC cell co-culture medium was detected using Griess reagent. **(D)** Gene expression levels in TAMs and LLC cells were analyzed by RT-qPCR. **(E, F)** Protein expression was analyzed by Western blotting. **(G, H)** Apoptosis in LLC cells was analyzed by FACS. Data were expressed as means ± SD and analyzed by unpaired Student’s *t*-test. *p < 0.05, **p < 0.01, and ***p < 0.001. Rel. mRNA level indicates relative mRNA level. LLC, Lewis lung cancer; FACS, fluorescence-activated cell sorting.

Based on the above *in vivo* and *in vitro* results, we next investigated the direct cytotoxic effect of PcrV-primed BMDMs on tumor cells by co-culturing these cells with LLC cells. The co-culture of BMDMs with tumor cells polarized normal BMDMs into TAMs. Hence, BMDMs are referred to as TAMs after their co-culture with tumor cells. We found that co-culture increased the rate of apoptosis among the tumor cells (**Figures 4A, B**). Considering that ROS and NO are critical mediators of cancer cell cytotoxicity, we also examined the levels of these factors in the co-culture system. Unexpectedly, PcrV treatment did not affect the levels of ROS either in TAMs or LLC cells (**Supplementary Figure 4**). In contrast, NO production was found to be higher in the PcrV/TAM-LLC cell co-culture medium than in that of the TAM-LLC cell group (**Figure 4C**). The levels of *iNos* (**Figure 4D**) and iNOS (**Figures 4E, F**) were also higher in both TAMs and LLC cells. The inhibition of NO production in PcrV/TAMs through *S*-methyl thiourea (SMT) treatment decreased apoptosis (**Figures 4A, B**) in LLC cells. The addition of the NO donor DETA-NONOate (DETA-NO) into a co-culture system in which TAMs had been pretreated with SMT led to an increase in NO levels in the culture medium (**Figure 4C**) while also promoting the apoptosis of LLC cells (**Figures 4G, H**) in the PcrV/TAM-LLC cell co-culture group, thus providing further evidence that PcrV-primed TAMs display enhanced NO-associated cytotoxicity against tumor cells.

Even though the levels of NO were similar among the three groups [LLC cells (**Supplementary Figure 5A**), TAM-LLC cell co-culture group (**Figure 4C**), and PcrV/TAM-LLC cell co-culture group (**Figure 4C**)] following the addition of DETA-NO to the culture medium, the increased concentrations of NO did not enhance the apoptosis rate of LLC cells cultured individually (**Supplementary Figure 5B**) or with BMDMs (**Figures 4G, H**) compared with that of the PcrV/TAM-LLC cell co-culture system, suggesting that the PcrV-mediated augmentation of TAM-associated cytotoxicity against cancer cells relies on a synergistic effect of NO and other factors generated by PcrV-primed TAMs.

In addition, to observe whether PcrV-primed TAMs can affect tumor growth and metastasis, BMDMs treated or not with PcrV were co-cultured with LLC cells, and the levels of related genes were analyzed. No differences in the expression levels of genes related to tumor growth and metastasis, such as *Cox2*, *Mmp9*, *Vegfa*, and *Hif1a*, were found in LLC cells that were co-cultured with PcrV-primed TAMs (**Figure 4D**).

### The Rewiring of Glycolysis in Tumor-Associated Macrophages by PcrV Enhances Their Nitric Oxide-Mediated Tumoricidal Activity

**Figure 5:**
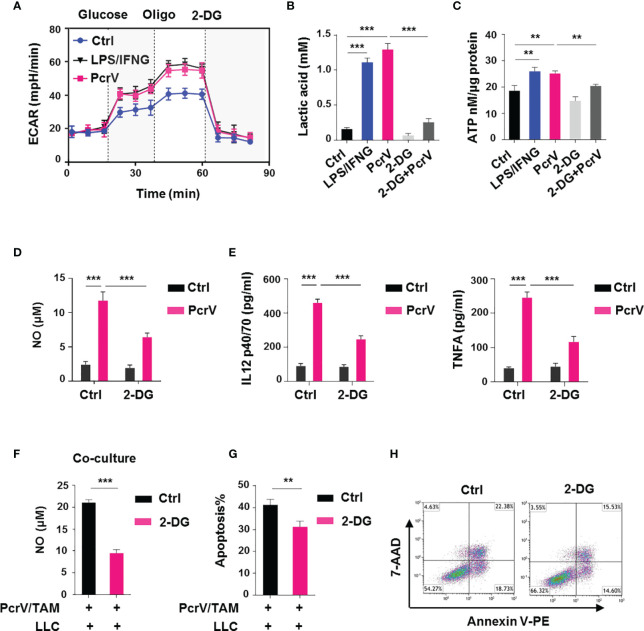
PcrV-induced stimulation of glycolysis augments tumor-associated macrophage (TAM)-mediated tumoricidal activity. To induce TAMs *in vitro*, bone marrow-derived macrophages (BMDMs) were cultured in 10% FBS/DMEM containing 20% (*v*/*v*) LLC cell culture supernatant for 24 h and then primed or not with PcrV (10 μg/ml) for another 24 h For the induction of an M1-like phenotype, TAMs were pulsed with LPS (10 ng/ml) and IFNG (10 ng/ml) for 24 h For the inhibition of glycolysis, TAMs were pretreated with 2-deoxy-d-glucose (2-DG; 2 mM) for 2 h followed by exposure to PcrV for another 24 h **(A)** Assay for the extracellular acidification rate (ECAR). **(B)** Measurement of the lactic acid level in the culture supernatant. **(C)** Assay for ATP levels in cell lysates. **(D)** The NO level in the culture supernatant was detected using Griess reagent. **(E)** IL12 p40/70 and TNFA levels in the culture supernatant were analyzed by ELISA. BMDMs pretreated with 2-DG (2 mM, 2 h) were primed with PcrV (10 μg/ml) for 24 h and then co-cultured with LLC cells for another 24 h **(F)** Detection of NO level in the culture medium. **(G, H)** The apoptosis of LLC cells was detected by FACS. Data were expressed as means ± SD and analyzed by unpaired Student’s *t*-test. **p < 0.01 and ***p < 0.001. Oligo, oligomycin; FBS, fetal bovine serum; DMEM, Dulbecco’s modified Eagle’s medium; LLC, Lewis lung cancer; FACS, fluorescence-activated cell sorting.

M1 macrophages are reported to rely on aerobic glycolysis for their energy supply (16), suggesting that the PcrV-mediated increase in TAM cytotoxic activity against cancer cells might involve the regulation of glycolysis. Similar to that seen with LPS/IFNG-primed M1-like TAMs, PcrV treatment increased the glycolysis-related ECAR (**Figure 5A**), lactic acid production (**Figure 5B**), and ATP generation (**Figure 5C**) in TAMs. The inhibition of glycolysis in TAMs by the application of 2-deoxy-d-glucose (2-DG) suppressed the PcrV-induced production of lactic acid (**Figure 5B**) and ATP (**Figure 5C**), as well as the levels of NO (**Figure 5D**), IL12 p40/70, and TNFA (**Figure 5E**), indicating that PcrV reprograms TAMs toward an M1 profile through increasing glycolysis. In addition, to examine the role of glycolysis in PcrV-induced, TAM-mediated cytotoxicity against cancer cells, BMDMs pretreated with 2-DG and PcrV were co-cultured with LLC cells. We found that 2-DG treatment decreased the PcrV-induced production of NO (**Figure 5F**) and the rate of apoptosis in LLC cells (**Figures 5G, H**), demonstrating that the enhancement of glycolysis by PcrV promotes the NO-associated tumoricidal activity of TAMs.

### PcrV-Mediated Activation of a PI3K/AKT/mTOR-Glycolysis-Nitric Oxide Feedback Loop Promotes Tumor-Associated Macrophage Repolarization and Cytotoxicity Against Cancer Cells

**Figure 6:**
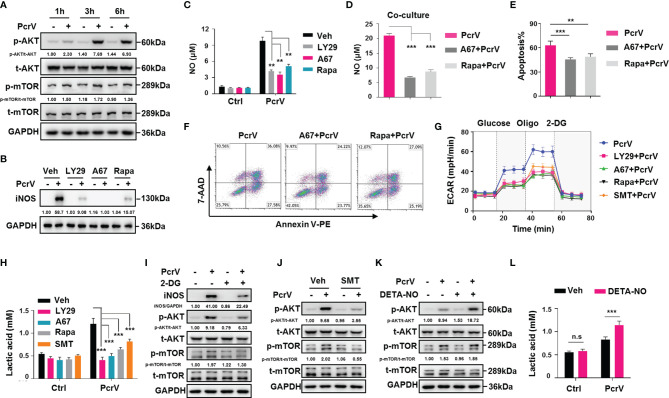
PcrV-mediated activation of the PI3K/AKT/mTOR-glycolysis-NO feedback loop reeducates tumor-associated macrophage (TAMs) and enhances TAM-mediated cytotoxicity against cancer cells. For the inhibition of the PI3K/AKT/mTOR axis, TAMs were pretreated with a PI3K (LY294002, 20 μM), AKT (A674563, 1 μM), or mTOR (rapamycin, 50 μM) inhibitor for 1 h and then primed with PcrV (10 μg/ml) for the indicated times. **(A, B)** Protein levels were analyzed by Western blotting. **(C)** The nitric oxide (NO) level in the culture supernatant (24 h) was detected using Griess reagent. Bone marrow-derived macrophages (BMDMs) pretreated with AKT or mTOR for 1 h were primed with PcrV (10 μg/ml) for 24 h and then co-cultured with LLC cells for another 24 h **(D)** Detection of NO levels in the culture medium. **(E, F)** The apoptosis of LLC cells was detected by FACS. TAMs pretreated with the PI3K/AKT/mTOR inhibitors (1 h), *S*-methyl thiourea (SMT; 0.5 mM, 2 h), or 2-deoxy-d-glucose (2-DG; 2 mM, 2 h) were primed with PcrV (10 μg/ml) for another 24 h **(G)** Extracellular acidification rate (ECAR) measurement. **(H)** Measurement of lactic acid levels in the culture supernatant. **(I, J)** Protein levels were analyzed by Western blotting. For increasing the NO concentration, TAM culture medium was supplemented with DETA-NO (20 μM) in combination or not with PcrV (1 μg/ml) for 24 h **(K)** Protein levels were analyzed by Western blotting. **(L)** Measurement of lactic acid level in the culture supernatant. Data were expressed as means ± SD and analyzed by unpaired Student’s *t*-test. **p < 0.01 and ***p < 0.001. Oligo, oligomycin; Veh, vehicle; LY29, LY294002; A67, A674563; Rapa, rapamycin; LLC, Lewis lung cancer; FACS, fluorescence-activated cell sorting.

Studies have shown that the PI3K/AKT**/**mTOR pathway is closely related to macrophage activation (17) as well as glycolysis in pulmonary fibrosis (18). To observe whether the PcrV-mediated regulation of TAM repolarization might involve this signaling pathway, we examined AKT and mTOR phosphorylation levels in TAMs treated or not with PcrV. The results revealed that PcrV treatment increased the phosphorylation levels of both proteins (**Figure 6A**) in TAMs. The treatment of PI3K, AKT, or mTOR with the corresponding inhibitor suppressed the levels of M1 markers in PcrV-treated TAMs, including that of *iNos* (**Supplementary Figure 6A**), iNOS (**Figure 6B**), NO (**Figure 6C**), IL12 p40/70, and TNFA (**Supplementary Figure 6B**) while increasing the levels of the M2 markers *Fn1*, *c-Myc*, and *Egr2* (**Supplementary Figure 6A**), suggesting that PcrV repolarizes TAMs into an M1 phenotype through the activation of the PI3K/AKT**/**mTOR signaling pathway. To further investigate whether the PI3K/AKT/mTOR signaling pathway is involved in the enhancement of the tumoricidal effect of TAMs elicited by PcrV, TAMs pretreated with the AKT or mTOR inhibitor plus PcrV were co-cultured with LLC cells. The suppression of AKT or mTOR activation reduced NO production in the co-culture medium (**Figure 6D**) and also decreased the levels of PcrV/TAM-induced apoptosis in LLC cells (**Figures 6E, F**).

We also investigated the cross-talk among PI3K/AKT**/**mTOR, glycolysis, and NO. The inhibition of PI3K, AKT, or mTOR led to impaired glycolysis-related ECAR and lactic acid production in PcrV-primed TAMs (**Figures 6G, H**). Inversely, the suppression of glycolysis by 2-DG led to impaired AKT and mTOR phosphorylation and iNOS expression (**Figure 6I**), suggesting that glycolysis positively regulates AKT/mTOR activation and NO generation in PcrV-primed TAMs. Given that NO promotes glycolysis in neurons (19) and also activates the PI3K/AKT axis in cancer cells (20, 21), we next examined the role of NO in triggering AKT/mTOR activation and glycolysis in PcrV-primed TAMs. Inhibiting NO generation in PcrV-primed TAMs with SMT treatment reduced AKT and mTOR phosphorylation (**Figure 6J**) as well as the ECAR (**Figure 6G**) and lactic acid levels (**Figure 6H**). Intriguingly, the exogenous administration of DETA-NO in combination with PcrV, but not DETA-NO alone, promoted AKT/mTOR activation in TAMs (**Figure 6K**) and upregulated lactic acid production (**Figure 6L**), indicating that NO-associated regulation of glycolysis and the AKT**/**mTOR signaling pathway in TAMs requires the involvement of other factors activated by PcrV. Collectively, these results indicated that the PcrV-mediated increase in the antitumoral effects of TAMs is associated with the activation of a PI3K/AKT**/**mTOR-glycolysis-NO feedback loop.

### TLR4/MyD88-Mediated Activation of the PI3K/AKT/mTOR-Glycolysis-Nitric Oxide Circuit Is Involved in Enhancing the Tumoricidal Activity of PcrV-Primed Tumor-Associated Macrophages

**Figure 7:**
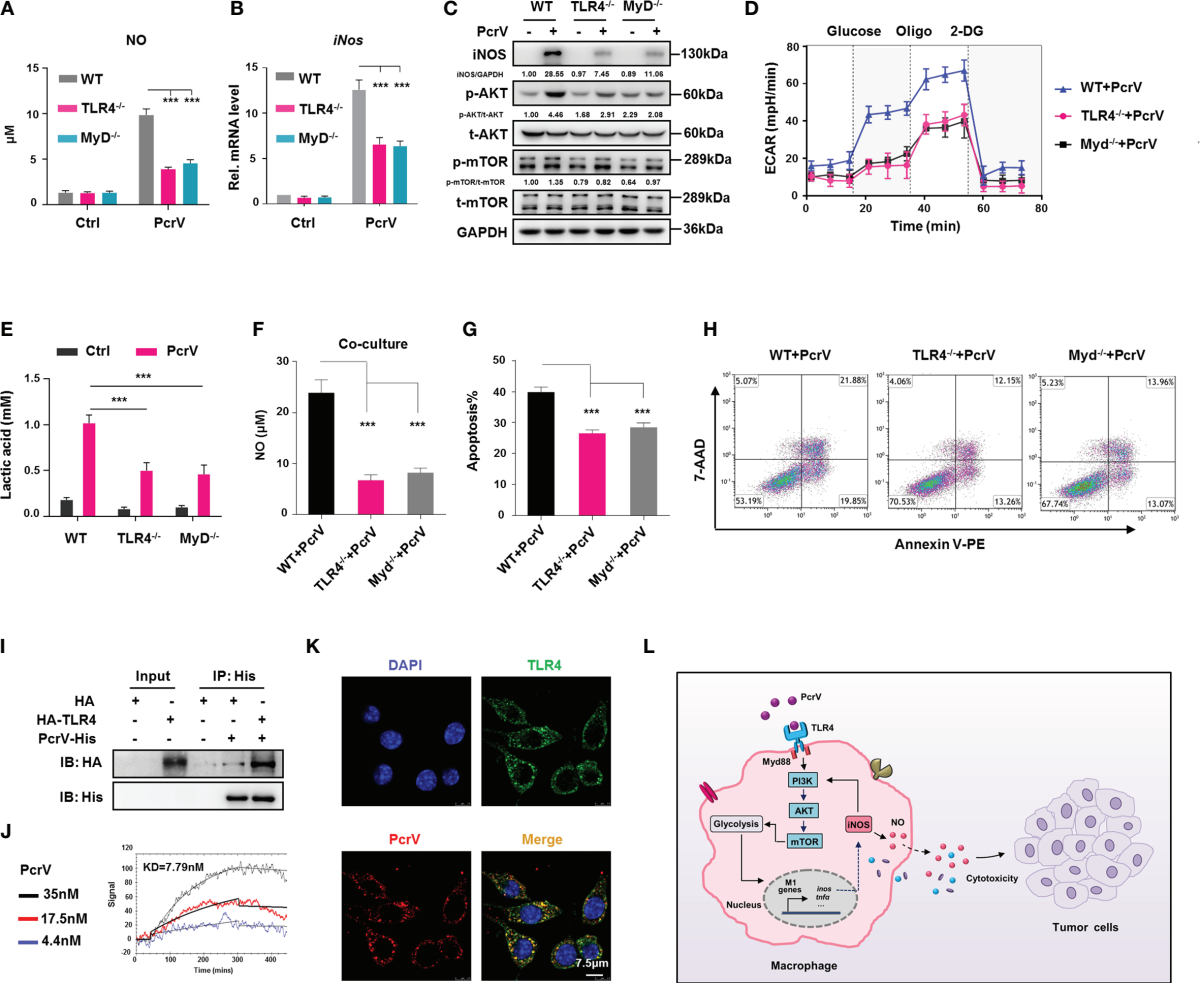
TLR4/MyD88-mediated activation of the PI3K/AKT/mTOR-glycolysis-NO circuit promotes PcrV-induced M1 polarization and tumor-associated macrophage (TAM)-mediated cytotoxicity. Wild type (WT), TLR4^−/−^, and MyD88^−/−^ TAMs were treated with PcrV (10 μg/ml) for 24 h **(A)** The nitric oxide (NO) level in the culture supernatant was measured using Griess reagent. **(B)** *iNos* expression levels were analyzed by RT-qPCR. **(C)** Protein levels were analyzed by Western blotting. **(D)** Assay for the extracellular acidification rate (ECAR). **(E)** Measurement of the lactic acid level in the culture supernatant. WT, TLR4^−/−^, and MyD88^−/−^ bone marrow-derived macrophages (BMDMs) were pretreated with PcrV (10 μg/ml) for 24 h and then co-cultured with LLC cells for another 24 h **(F)** Detection of NO level in the culture medium. **(G, H)** The apoptosis of LLC cells was detected by FACS. **(I)** The lysates of HEK293T cells overexpressing recombinant human TLR4 were incubated with purified PcrV protein. A co-immunoprecipitation (Co-IP) assay and Western blotting were performed to detect the interaction between PcrV and TLR4. **(J)** Direct interaction between TLR4 and PcrV was analyzed using surface plasmon resonance (SPR). **(K)** PcrV/TLR4 co-colonization on Raw264.7 cells was visualized using immunofluorescence (IF) staining. **(L)** Schematic diagram of the molecular mechanism by which PcrV reeducates TAMs and enhances TAM-mediated antitumoral activity. Data were expressed as means ± SD and analyzed by unpaired Student’s *t*-test. ***p < 0.001. MyD indicates MyD88. TLR4, Toll-like receptor 4; MyD88, myeloid differentiation factor 88; LLC, Lewis lung cancer; FACS, fluorescence-activated cell sorting.

TLR4, a key pattern recognition receptor (PPR), is abundantly expressed on immune cells, where it functions in the recognition of pathogen-associated molecular patterns (PAMPs). Following pathogen recognition, TLR4 activates host immune response through both MyD88-dependent and MyD88-independent mechanisms (22). Given that PcrV is a needle tip protein component of the T3SS of *P. aeruginosa*, it seems likely that it can interact with host PPRs and thus activate immune responses. To address this possibility, we investigated the role of the TLR4/MyD88 signaling pathway in PcrV-mediated regulation of TAM polarization. Compared with WT PcrV-primed TAMs, TLR4^−/−^ or MyD88^−/−^ PcrV-primed TAMs showed reduced levels of NO (**Figure 7A**), *iNos* (**Figure 7B**), iNOS (**Figure 7C**), IL12 p40/70, and TNFA (**Supplementary Figure 7A**) but higher levels of *c-Myc*, *Egr2*, and *Fn1* (**Supplementary Figure 7B**), suggesting that PcrV skews the TAM phenotype toward an M1 profile through the TLR4-MyD88 signaling pathway.

As it has been reported that the PI3K/AKT/mTOR pathway is under the control of the TLR4/MyD88 signaling axis (23), we next examined PI3K/AKT/mTOR pathway activation in TLR4^−/−^ or MyD88^−/−^ PcrV-primed TAMs. The results showed that the PcrV-induced activation of AKT and mTOR was impaired in TLR4^−/−^ or MyD88^−/−^ TAMs, respectively (**Figure 7C**). Analyses of glycolysis-related factors demonstrated that the PcrV-induced increase in the ECAR (**Figure 7D**) and lactic acid production (**Figure 7E**) was reduced in TLR4^−/−^ and MyD88^−/−^ TAMs.

We further analyzed the role of the TLR4/MyD88 axis in the PcrV-mediated regulation of the tumoricidal effect of TAMs. The co-culture of LLC cells with PcrV-primed TLR4^−/−^ or MyD88^−/−^ TAMs, but not WT TAMs, reduced NO production in the culture medium (**Figure 7F**) as well as the levels of apoptosis in LLC cells (**Figures 7G, H**). In addition, the LLC tumor-bearing mice in which endogenous macrophages were depleted by clodronate liposomes were intratumorally injected with WT, TLR4^−/−^, or MyD88^−/−^ BMDMs primed or not with PcrV (**Supplementary Figure 8A**). The result showed that treatment with PcrV-primed WT BMDMs, but not TLR4^−/−^ or MyD88^−/−^ BMDMs, decreased tumor growth (**Supplementary Figure 8B**) and weight (**Supplementary Figure 8C**). Collectively, these results indicated that the TLR4/MyD88 signaling axis participates in the PcrV-mediated modulation of the PI3K/AKT/mTOR-glycolysis-NO circuit and the tumoricidal effect of TAMs.

### Direct Interaction Between PcrV and TLR4 Is Required for the PcrV-Mediated Reeducation of Tumor-Associated Macrophages

To further explore whether PcrV alters TAM polarization through interaction with TLR4, we performed immunoprecipitation by incubating purified PcrV protein with the lysate of HEK293T cells overexpressing recombinant human TLR4 protein. The result showed a successful pull-down of TLR4 protein by PcrV (**Figure 7I**). SPR analysis using purified protein further revealed a direct interaction between TLR4 and PcrV (**Figure 7J**). Moreover, IF staining result showed that PcrV colocalized with TLR4 in Raw264.7 macrophages (**Figure 7K**). In addition, to examine the effect of PcrV–TLR4 interaction on PcrV-mediated regulation of TAMs, we blocked TLR4 using an antibody, and we found that the PcrV-induced production of IL12 p40/70, TNFA (**Supplementary Figure 7C**), and NO (**Supplementary Figure 7D**) in TAMs was reduced, indicating that PcrV-mediated regulation of TAM repolarization involves direct interaction between PcrV and TLR4.

## Discussion

TAMs can constitute up to 50% of a tumor mass, forming the major component of the tumor immune cell infiltrate. In the TME, M2-like TAMs promote tumor cell growth, invasion, and metastasis, angiogenesis, and infiltration of immune-suppressive cells, while suppressing antitumoral immune surveillance (24). In addition, TAMs are known to increase resistance to standard-of-care therapeutics, including chemotherapy, irradiation, and angiogenic inhibitors (25). In contrast, tumoricidal M1-like macrophages, which express high levels of TNF, iNOS, and MHC molecules and low levels of ARG1, IL-10, CD163, and CD206 (26), play roles in reversing immune suppression in the TME and enhancing macrophage- or T cell-mediated killing of cancer cells. In tumor-initiating conditions, macrophages exhibit antitumoral activity; however, once tumors are established, macrophages are reeducated into a protumoral phenotype (27), likely because macrophages are highly plastic in terms of functional reprogramming in response to stimuli in the TME, such as hypoxia, cytokines, and chemokines, as well as in response to varied interactions with components of the extracellular matrix (24, 28). Hence, approaches that can potentiate TAM reprogramming into a tumoricidal M1 phenotype show great promise in cancer immunotherapy. In this study, we found that intratumoral injection of PcrV reduces tumor growth and increases the rate of apoptosis in tumor tissues by reeducating TAMs into an M1 profile characterized by the elevated expression of M1 markers (e.g., iNOS, MHCII, and CD86) and reduced levels of M2 markers (e.g., ARG1 and CD206). Additionally, we found that PcrV-induced NO production increased M1 TAM-mediated cytotoxicity against cancer cells *in vitro*. These results highlight the feasibility of utilizing *P. aeruginosa*-derived PcrV in immunomodulation and cancer therapy. Similarly, other well-known bacterial molecules, such as MPT63 (*M. bovis*), arginine deiminase (*Mycoplasma arginine*), lapidated-azurin (*Neisseria meningitidis*), and azurin (*P. aeruginosa*), have been reported as potential anticancer drugs (29). However, factors such as bacterial endotoxin, manufacturing technique, protein stability, administration mode, and biosafety still need to be addressed when utilizing bacteria-derived proteins as antitumoral drugs (29, 30).

TLR4, which is expressed on immune cells such as macrophages, dendritic cells (DCs), T cells, neutrophils, and epithelial cells, is one of the major sensors of PAMPs/damage-associated molecular patterns (DAMPs) that activate adaptive immune responses. The activation of TLR4 on multiple immune cells, such as T cells and DCs, represents a powerful means of suppressing tumor growth (31–33). In addition, several studies have reported that TLR4-dependent TAM reprogramming into an M1 profile reduces tumor growth (3, 34). In this study, we found that PcrV directly interacts with TLR4 expressed on macrophages, which induces TAM M1 polarization and enhances TAM-mediated killing of tumor cells *via* the TLR4/MyD88 signaling pathway. Surprisingly, the activation of TLR4 expressed on tumor cells has been proposed to also promote tumor development by increasing the production of oncogenic mediators (e.g., COX2, IL6, VEGF, and TGFβ) *via* the activation of proinflammatory and protumoral signaling pathways, such as the NF-κB, MAPK, and COX2/PGE2 pathways (35, 36). However, in this study, LLC cells treated with PcrV or PcrV-primed macrophages did not show significantly increased production of oncogenic mediators or metastatic ability, indicating that PcrV exerts its antitumoral effects mainly through the targeting of TAMs. The differences observed between macrophages and cancer cells might be partially attributable to differences in the expression levels of TLR4 or other accessory molecules expressed on these cells.

The PI3K/AKT/mTOR axis, which is regulated by TLR4, functions as a critical signaling pathway in modulating macrophage polarization. The inhibition of the PI3K/AKT/mTOR or the PI3K/AKT signaling pathway in macrophages suppresses M1 macrophage polarization (17) and polarizes M1 macrophages into an M2-like phenotype (37). In addition, both the PI3K/AKT/mTOR and AKT/mTOR axes are reported to be involved in enhancing glycolysis in tumor cells (38) and TAMs (39), which, in turn, promotes tumor cell survival and proliferation. In line with these findings, our results showed that PcrV-induced activation of the PI3K/AKT/mTOR pathway promotes both M1 polarization and glycolysis in TAMs. However, in contrast to previously reported results, we found that PcrV-primed TAMs exert cytotoxic effects against cancer cells rather than protumoral activity. These discrepant results might be partially explained by the contrasting (cytoprotective/cytotoxic) effects elicited by NO.

NO, a gas with diverse biological activities produced from arginine by NO synthases, has long been known as a cytotoxic agent that can directly induce the apoptosis of cancer cells (40), as well as enhance radiation-/chemotherapeutic agent-induced apoptosis of cancer cells (41, 42). In our study, we found that the co-culture of LLC cells with PcrV-primed TAMs leads to a marked increase in NO levels in the co-culture medium as well as iNOS expression in LLC cells, which, in turn, induces LLC cell apoptosis in a NO-dependent manner. Intriguingly, the supplementation of exogenous NO to LLC cells or TAM-LLC cell co-culture medium did not enhance LLC cell apoptosis, whereas increased levels of LLC cell apoptosis were seen when exogenous NO was applied to co-cultures of LLC cells and PcrV-primed TAMs. These results indicate that NO-associated cytotoxicity relies on the involvement of other mediators supplied by macrophages following PcrV priming. In contrast to the NO-associated cytotoxic effects, NO has been suggested to enhance tumor cell growth and metastasis (43) and help tumor cells resist chemotherapeutic agent-induced apoptosis (44). These contradictory effects might be due to the concentration and duration of exposure to NO encountered by cancer cells (45). NO is cytoprotective at low/physiological levels or with short exposure time but is cytotoxic when produced at high concentrations or under long periods of exposure (46). We and others have reported that PI3K/AKT/mTOR activation and glycolysis rewire TAMs into an M1-polarized phenotype that, in turn, promotes NO production (47, 48). Here, we further found that NO, in combination with PcrV, activates the PI3K/AKT/mTOR-glycolysis signaling pathway in TAMs, resulting in the formation of a PI3K/AKT/mTOR-glycolysis-NO feedback loop that increases NO generation and, consequently, NO-associated cytotoxicity against cancer cells (**Figure 7L**).

Combined, our findings revealed a tumoricidal role for PcrV mediated by the reeducation of TAMs into a tumoricidal M1 phenotype through the modulation of a PI3K/AKT/mTOR-glycolysis-NO feedback loop *via* a direct interaction with TLR4. Our findings provide an alternative therapeutic approach for inhibiting tumor development.

## Data Availability Statement

The raw data supporting the conclusions of this article will be made available by the authors, without undue reservation.

## Ethics Statement

The animal study was reviewed and approved by Army Medical University of China.

## Author Contributions

KZ, XHe, and HY designed the experiment. KZ, HY, YB, and XHu analyzed the data and wrote the manuscript. YB, HY, JQ, JX, QD, XW, YL, HS, RX, LJ, QL, DL, HZ, LZ, QC, and JP performed the experiments. All authors contributed to the article and approved the submitted version.

## Funding

This project was supported by grants from the National Natural Science Foundation of China (No. 31872634 and No. 31700129), Basic Foundation of Army Medical University (2021-2018-067) and Special Financial Aid to Postdoctoral Research Fellow in Chongqing.

## Conflict of Interest

The authors declare that the research was conducted in the absence of any commercial or financial relationships that could be construed as a potential conflict of interest.

## Publisher’s Note

All claims expressed in this article are solely those of the authors and do not necessarily represent those of their affiliated organizations, or those of the publisher, the editors and the reviewers. Any product that may be evaluated in this article, or claim that may be made by its manufacturer, is not guaranteed or endorsed by the publisher.

## Supplementary Material

Supplementary Figure 1 PcrV does not exert apparent cytotoxicity and protumoral effect. LLC cells and TAMs were treated with PcrV at the indicated concentrations for 48 h. **(A)** Cell viability was analyzed by CCK8 assay. **(B)** Gene expression levels of LLC cells were analyzed by RT-qPCR. **(C)** Protein levels of LLC cells were detected by Western blotting. **(D, E)** Metastatic ability of LLC cells treated with or without PcrV (10 μg/mL) for 24 or 48 h was analyzed. Data were expressed as means ± SD and analyzed by one-way ANOVA **(A, B)** or unpaired Student’s t-test **(E)**. n.s (No significance); Rel. mRNA level (Relative mRNA level).

Supplementary Figure 2 PcrV treatment does not affect the percentages of CD4+ and CD8+ T cells, NK cells and MDSCs in tumor tissues of LLC tumor-bearing mice. FACS analysis of the percentages of CD4+ T (CD45+CD4+), CD8+ T (CD45+CD8+) **(A, B)**, NK cells (CD45+NK1.1+) **(C, D)**, MDSCs (CD45+CD11b+Gr1+) **(E, F)** and TAMs (CD45+F4/80+) **(G, H)** in tumor tissues of LLC tumor-bearing mice treated with PBS or PcrV. Data were expressed as means ± SD (n = 6) and analyzed by unpaired Student’s t-test.

Supplementary Figure 3 PcrV skews TAMs towards an M1 phenotype in vitro. TAMs were induced by treating BMDMs with 10% FBS/DMEM containing 20% (v/v) LLC cell culture supernatant for 24 h. TAMs were then primed with or without PcrV (10 μg/mL) for another 24 h. **(A, B)** Gene expression levels were analyzed by RT-qPCR. **(C)** iNOS protein level was analyzed by Western blotting. **(D)** NO production in culture supernatant was measured by Griess reagent. **(E)** IL12 p40/70 and TNFA levels in culture supernatants were analyzed by ELISA. **(F, G)** Cell surface markers, MHCII and CD86, were analyzed by FACS. Data were expressed as means ± SD and analyzed by unpaired Student’s t-test. **P < 0.01 and ***P < 0.001.

Supplementary Figure 4 PcrV does not affect ROS production in TAMs or LLC cells. BMDMs were pretreated with or without PcrV (10 μg/mL) for 24 h. Then, the supernatants were discarded, and cells were cocultured with LLC cells for 24 h by using Transwell plate (0.4-μm pore). TAMs and LLC cells were separately harvested and stained with H2DCFDA at RT for 30 min. Intracellular ROS in TAMs **(A, B)** and LLC cells **(C, D)** were measured by FACS. Data were expressed as means ± SD and analyzed by unpaired Student’s t-test.

Supplementary Figure 5 NO does not enhance the apoptosis rate of LLC cells cultured individually. The NO donor, DETA-NO, was supplemented into the culture medium of LLC cells, and then cells were incubated at 37°C with 5% CO2 for 24 h. **(A)** NO production in culture supernatant was measured by Griess reagent. **(B)** Apoptosis was analyzed by FACS. Data were expressed as means ± SD, and analyzed by one-way ANOVA. **P < 0.01 and ***P < 0.001.

Supplementary Figure 6 PI3K/AKT/mTOR signaling pathway is involved in PcrV-mediated regulation of TAM polarization. TAMs were pretreated with the inhibitor of PI3K (LY294002, 20 μM), AKT (A674563, 1 μM) or mTOR (Rapamycin, 50 μM) for 1 h. Then, the cells were primed with PcrV (10 μg/mL) for another 24 h. **(A)** Gene expression levels were analyzed by RT-qPCR. **(B)** IL12 p40/70 and TNFA levels in culture supernatants were detected by ELISA. Data were expressed as means ± SD and analyzed by unpaired Student’s t-test. **P < 0.01 and ***P < 0.001. Veh (Vehicle), LY29 (LY294002), A67 (A674563) and Rapa (Rapamycin).

Supplementary Figure 7 PcrV regulates TAM polarization via the TLR4-MyD88 signaling pathway. WT, TLR4-/- and MyD88-/- TAMs were treated with PcrV (10 μg/mL) for 24 h. **(A)** IL12 p40/70 and TNFA levels in culture supernatants were analyzed by ELISA. **(B)** Gene expression levels were analyzed by RT-qPCR. TAMs were pretreated with TLR4 antibody (5 μg/mL)at 37°C for 1 h to block TLR4 expressed on TAMs. Then, the cells were primed with PcrV (10 μg/mL) for 24 h. **(C)** Detection of IL12 p40/70 and TNFA levels in culture supernatants. **(D)** NO level in culture supernatant was measured by Griess reagent. Data were expressed as means ± SD and compared by unpaired Student’s t-test. *P < 0.05, **P < 0.01 and ***P < 0.001. Ab indicates antibody.

Supplementary Figure 8 PcrV-primed BMDMs, but not TLR4^-/-^ or MyD88^-/-^ BMDMs, suppress tumor growth. **(A)** Schematic of the mouse models used. Tumor growth **(B)** and weight **(C)** were measured in LLC cell-derived tumor-bearing mice treated with WT, TLR4^-/-^ or MyD88^-/-^ BMDMs primed or not with PcrV. Data were expressed as means ± SEM [**(B)**, n = 4] or means ± SD [**(C)**, n = 4], and were analyzed by two-way ANOVA **(B)** or unpaired Student’s t-test **(C)**. **P < 0.01.
