# Supplementary figures and images for: Pseudomonas aeruginosa PcrV Enhances the Nitric Oxide-Mediated Tumoricidal Activity of Tumor-Associated Macrophages via a TLR4/PI3K/AKT/mTOR-Glycolysis-Nitric Oxide Circuit
Source: Front Oncol. 2021 Nov 25;11:736882. doi: 10.3389/fonc.2021.736882 (PMC8654729; doi:10.3389/fonc.2021.736882)

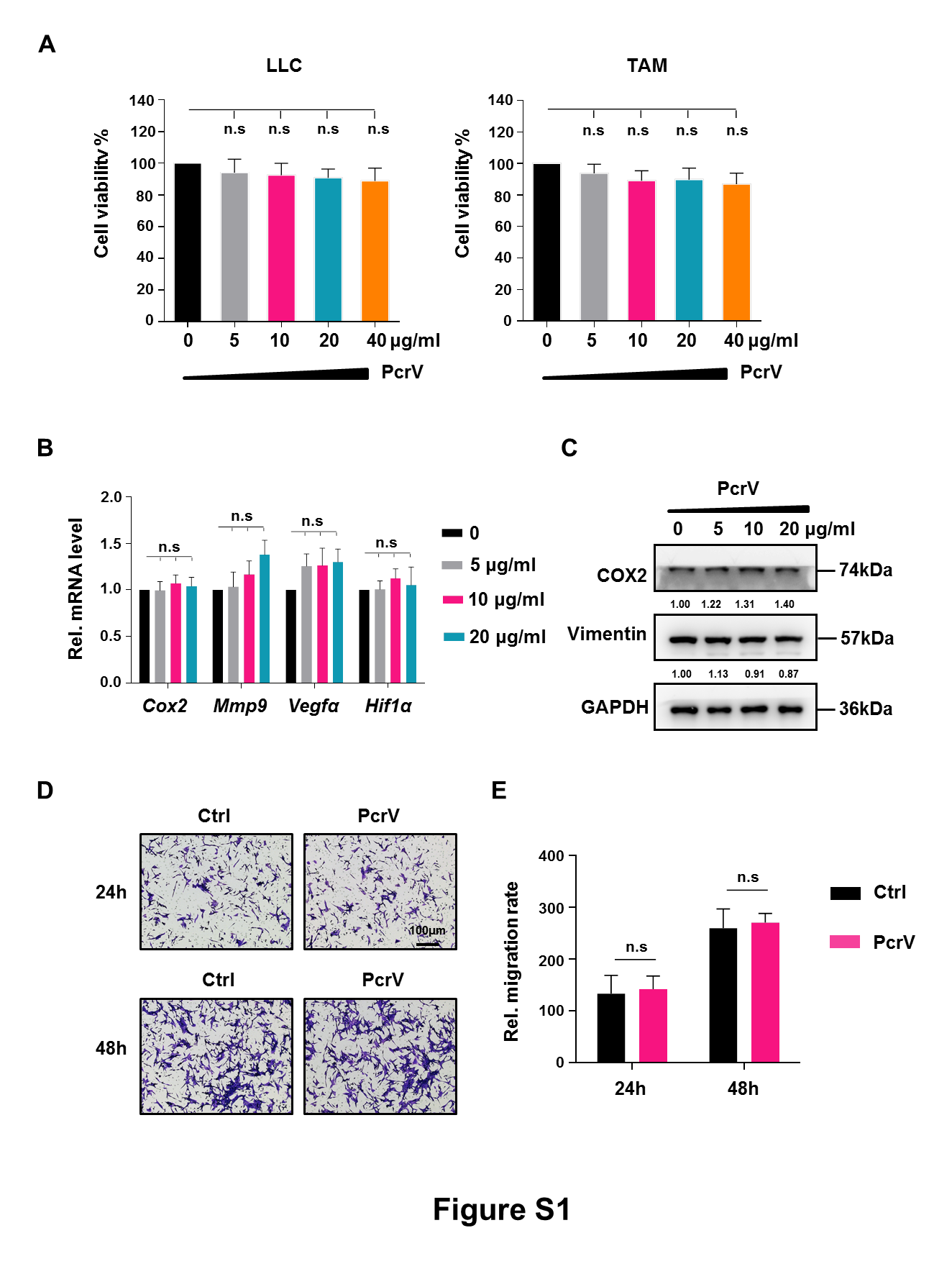

Supplement: Supplementary Figure 1 — PcrV does not exert apparent cytotoxicity and protumoral effect. LLC cells and TAMs were treated with PcrV at the indicated concentrations for 48 h. (A) Cell viability was analyzed by CCK8 assay. (B) Gene expression levels of LLC cells were analyzed by RT-qPCR. (C) Protein levels of LLC cells were detected by Western blotting. (D, E) Metastatic ability of LLC cells treated with or without PcrV (10 μg/mL) for 24 or 48 h was analyzed. Data were expressed as means ± SD and analyzed by one-way ANOVA (A, B) or unpaired Student’s t-test (E). n.s (No significance); Rel. mRNA level (Relative mRNA level). [file Image_1.tif]

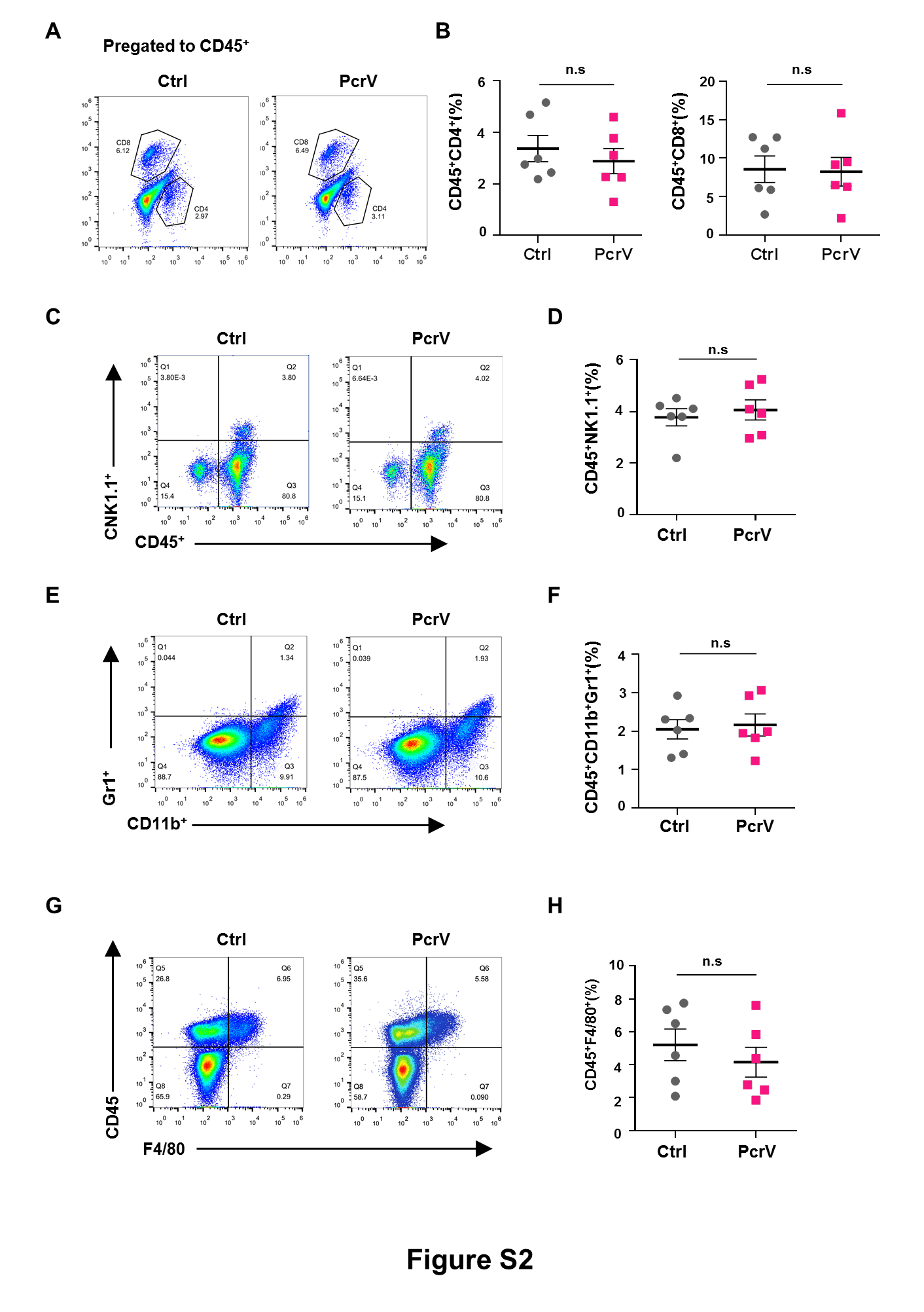

Supplement: Supplementary Figure 2 — PcrV treatment does not affect the percentages of CD4+ and CD8+ T cells, NK cells and MDSCs in tumor tissues of LLC tumor-bearing mice. FACS analysis of the percentages of CD4+ T (CD45+CD4+), CD8+ T (CD45+CD8+) (A, B), NK cells (CD45+NK1.1+) (C, D), MDSCs (CD45+CD11b+Gr1+) (E, F) and TAMs (CD45+F4/80+) (G, H) in tumor tissues of LLC tumor-bearing mice treated with PBS or PcrV. Data were expressed as means ± SD (n = 6) and analyzed by unpaired Student’s t-test. [file Image_2.tif]

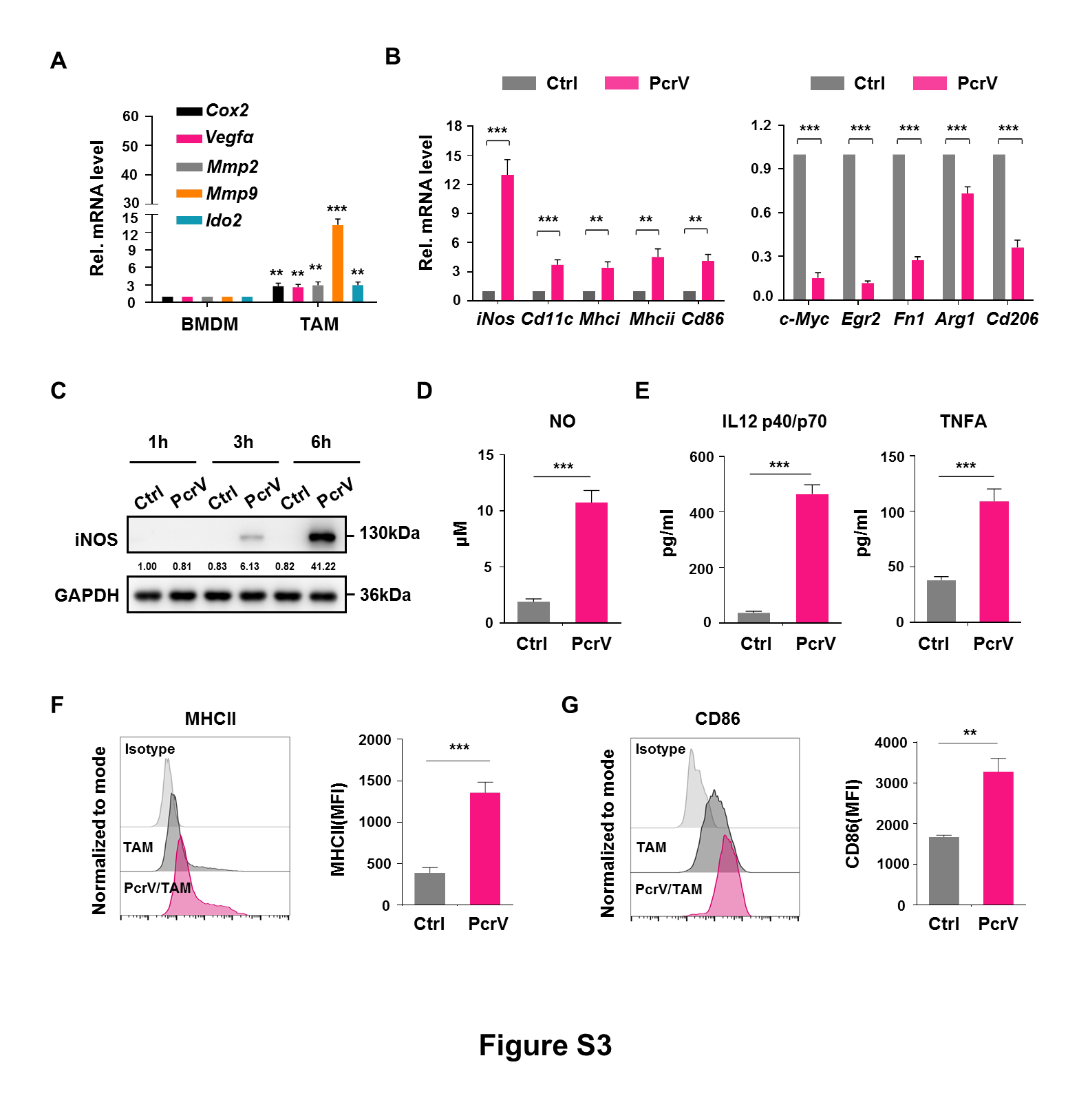

Supplement: Supplementary Figure 3 — PcrV skews TAMs towards an M1 phenotype in vitro. TAMs were induced by treating BMDMs with 10% FBS/DMEM containing 20% (v/v) LLC cell culture supernatant for 24 h. TAMs were then primed with or without PcrV (10 μg/mL) for another 24 h. (A, B) Gene expression levels were analyzed by RT-qPCR. (C) iNOS protein level was analyzed by Western blotting. (D) NO production in culture supernatant was measured by Griess reagent. (E) IL12 p40/70 and TNFA levels in culture supernatants were analyzed by ELISA. (F, G) Cell surface markers, MHCII and CD86, were analyzed by FACS. Data were expressed as means ± SD and analyzed by unpaired Student’s t-test. **P < 0.01 and ***P < 0.001. [file Image_3.tif]

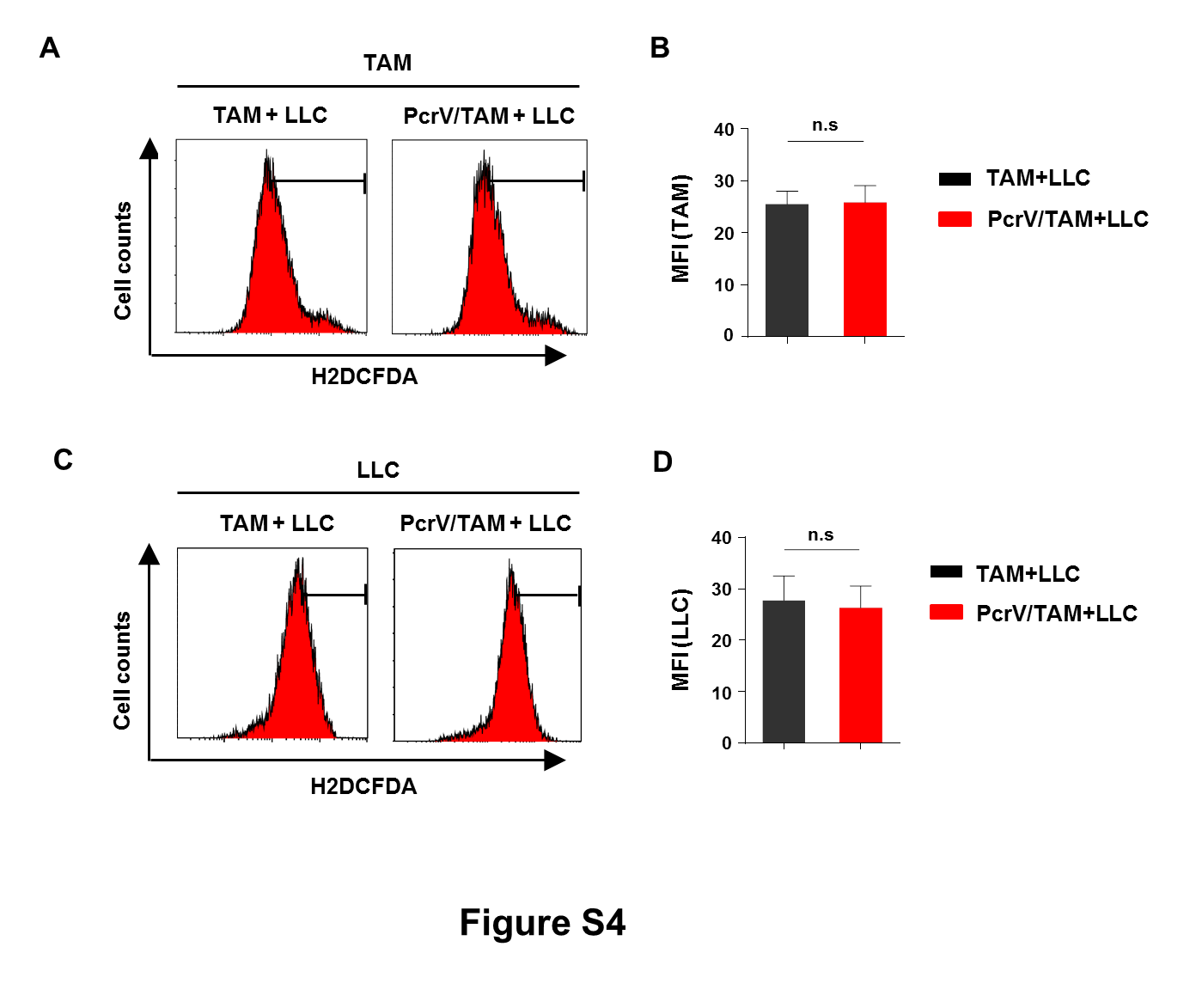

Supplement: Supplementary Figure 4 — PcrV does not affect ROS production in TAMs or LLC cells. BMDMs were pretreated with or without PcrV (10 μg/mL) for 24 h. Then, the supernatants were discarded, and cells were cocultured with LLC cells for 24 h by using Transwell plate (0.4-μm pore). TAMs and LLC cells were separately harvested and stained with H2DCFDA at RT for 30 min. Intracellular ROS in TAMs (A, B) and LLC cells (C, D) were measured by FACS. Data were expressed as means ± SD and analyzed by unpaired Student’s t-test. [file Image_4.tif]

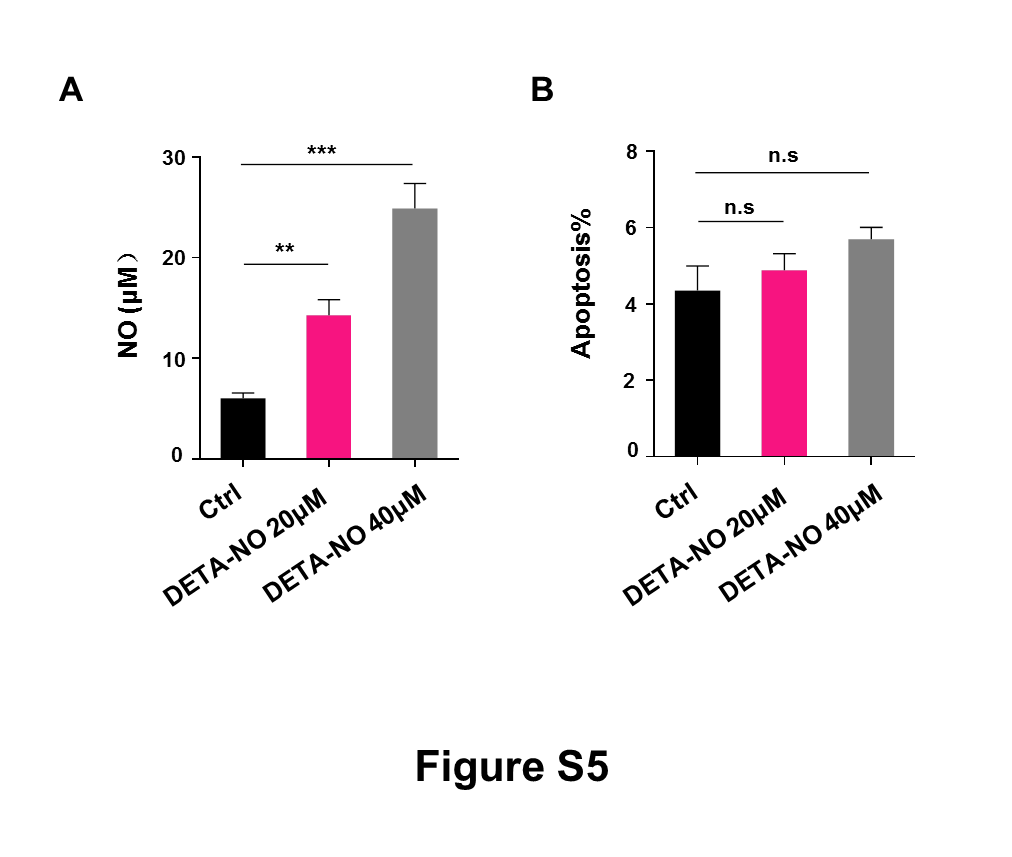

Supplement: Supplementary Figure 5 — NO does not enhance the apoptosis rate of LLC cells cultured individually. The NO donor, DETA-NO, was supplemented into the culture medium of LLC cells, and then cells were incubated at 37°C with 5% CO2 for 24 h. (A) NO production in culture supernatant was measured by Griess reagent. (B) Apoptosis was analyzed by FACS. Data were expressed as means ± SD, and analyzed by one-way ANOVA. **P < 0.01 and ***P < 0.001. [file Image_5.tif]

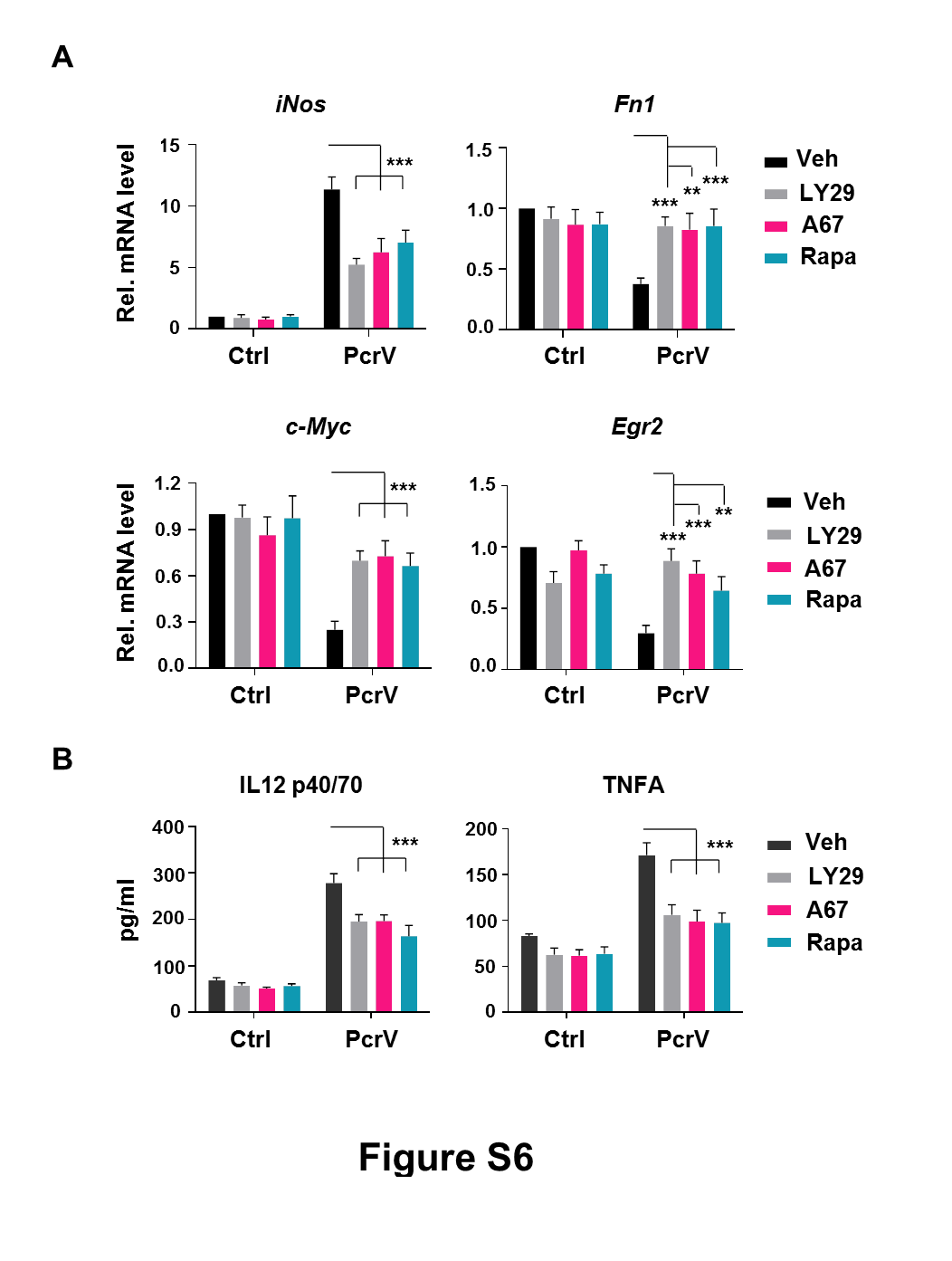

Supplement: Supplementary Figure 6 — PI3K/AKT/mTOR signaling pathway is involved in PcrV-mediated regulation of TAM polarization. TAMs were pretreated with the inhibitor of PI3K (LY294002, 20 μM), AKT (A674563, 1 μM) or mTOR (Rapamycin, 50 μM) for 1 h. Then, the cells were primed with PcrV (10 μg/mL) for another 24 h. (A) Gene expression levels were analyzed by RT-qPCR. (B) IL12 p40/70 and TNFA levels in culture supernatants were detected by ELISA. Data were expressed as means ± SD and analyzed by unpaired Student’s t-test. **P < 0.01 and ***P < 0.001. Veh (Vehicle), LY29 (LY294002), A67 (A674563) and Rapa (Rapamycin). [file Image_6.tif]

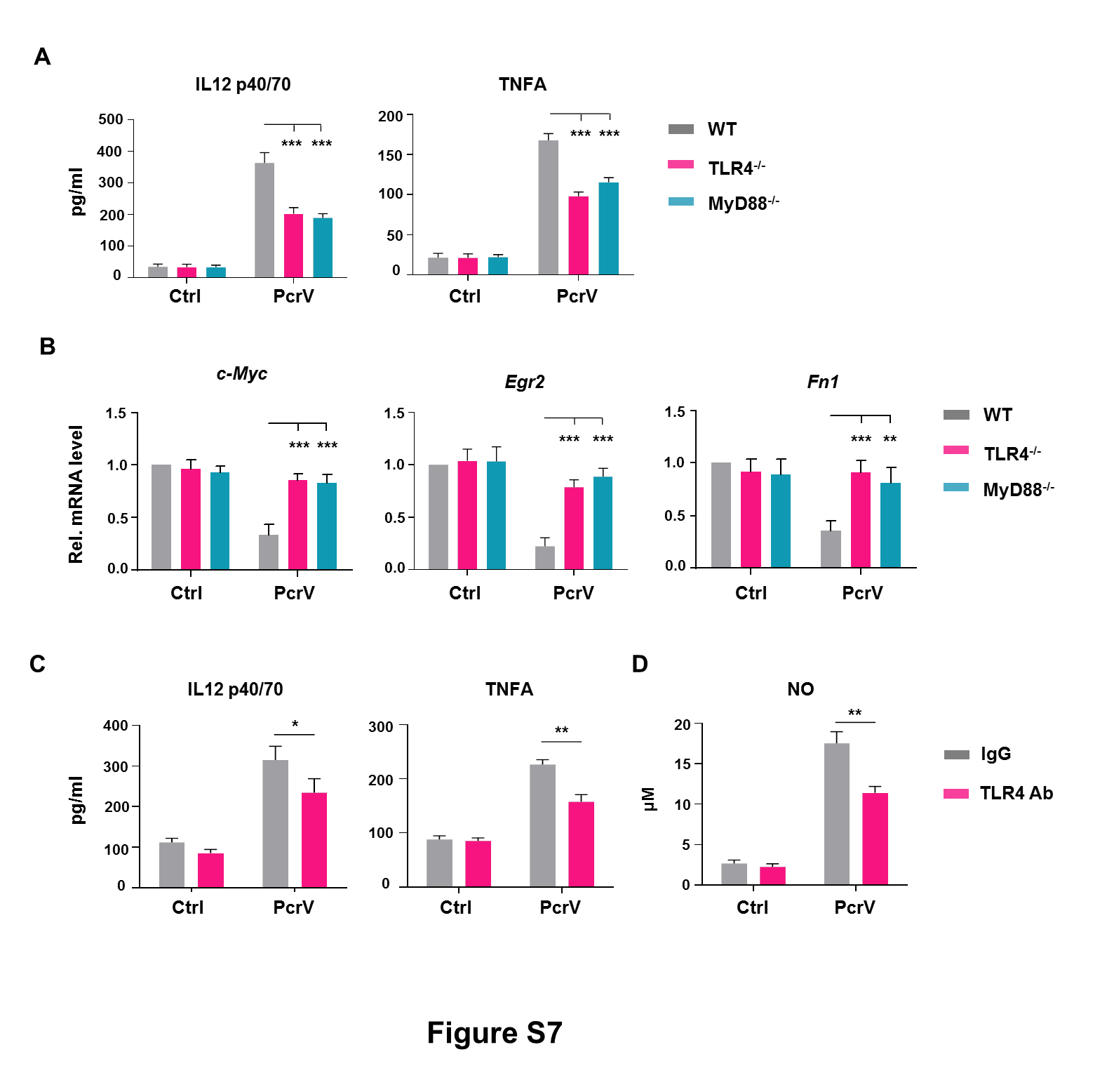

Supplement: Supplementary Figure 7 — PcrV regulates TAM polarization via the TLR4-MyD88 signaling pathway. WT, TLR4-/- and MyD88-/- TAMs were treated with PcrV (10 μg/mL) for 24 h. (A) IL12 p40/70 and TNFA levels in culture supernatants were analyzed by ELISA. (B) Gene expression levels were analyzed by RT-qPCR. TAMs were pretreated with TLR4 antibody (5 μg/mL)at 37°C for 1 h to block TLR4 expressed on TAMs. Then, the cells were primed with PcrV (10 μg/mL) for 24 h. (C) Detection of IL12 p40/70 and TNFA levels in culture supernatants. (D) NO level in culture supernatant was measured by Griess reagent. Data were expressed as means ± SD and compared by unpaired Student’s t-test. *P < 0.05, **P < 0.01 and ***P < 0.001. Ab indicates antibody. [file Image_7.tif]

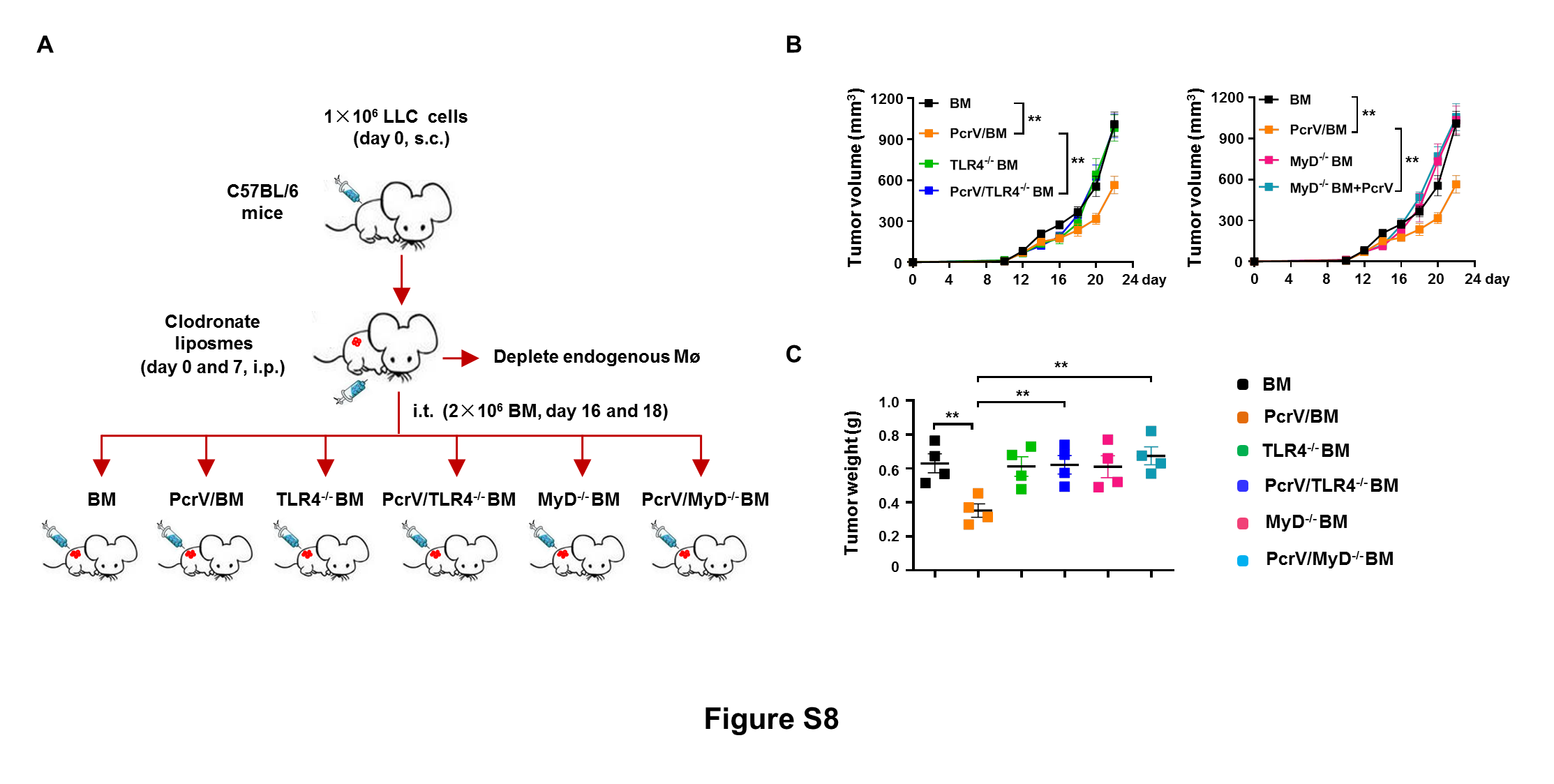

Supplement: Supplementary Figure 8 — PcrV-primed BMDMs, but not TLR4-/- or MyD88-/- BMDMs, suppress tumor growth. (A) Schematic of the mouse models used. Tumor growth (B) and weight (C) were measured in LLC cell-derived tumor-bearing mice treated with WT, TLR4-/- or MyD88-/- BMDMs primed or not with PcrV. Data were expressed as means ± SEM [(B), n = 4] or means ± SD [(C), n = 4], and were analyzed by two-way ANOVA (B) or unpaired Student’s t-test (C). **P < 0.01. [file Image_8.tif]
