# Supplementary material for: Pseudomonas aeruginosa PcrV Enhances the Nitric Oxide-Mediated Tumoricidal Activity of Tumor-Associated Macrophages via a TLR4/PI3K/AKT/mTOR-Glycolysis-Nitric Oxide Circuit
Source: Front Oncol. 2021 Nov 25;11:736882. doi: 10.3389/fonc.2021.736882 (PMC8654729; doi:10.3389/fonc.2021.736882)
Supplement: Supplementary file 9 [file Table_1.docx]

Supplementary Table 1. Primers used in this study

| inos-FP | 5' gttctcagcccaacaatacaaga 3' |
| --- | --- |
| inos-RP | 5' gtggacgggtcgatgtcac 3' |
| cd11c-FP | 5' agagccagaacttcccaact 3' |
| cd11c-RP | 5' ctacccgagccatcaatc 3' |
| mhci-FP | 5' agagagactcagggcctacc 3' |
| mhci-RP | 5' ccaggtcagggtgatgtcag 3' |
| mhcii-FP | 5' cataggtgcctacgtggtcg 3' |
| mhcii-RP | 5' accaagtgggagggaggaat 3' |
| cd86-FP | 5' ttacggaagcacccacgatg 3' |
| cd86-RP | 5' cggcagatatgcagtcccat 3' |
| cymc-FP | 5' ttcctttgggcgttggaaac 3' |
| cymc-RP | 5' gggctgtacggagtcgtagt 3' |
| egr2-FP | 5' ccccaatggtgaactgggag 3' |
| egr2-RP | 5' gcgaagctactcggatacgg 3' |
| fn1-FP | 5' accgacgaagagcccttaca 3' |
| fn1-RP | 5' atggcaccattcagcgttgc 3' |
| arg1-FP | 5' tgggaagacagcagagga 3' |
| arg1-RP | 5' tcagtccctggcttatgg 3' |
| cd206-FP | 5' gcaagtgatttggaggct 3' |
| cd206-RP | 5' ataggaaacgggagaacc 3' |
| cox2-FP | 5' tgcactatggttacaaaagctgg 3' |
| cox2-RP | 5' tcaggaagctccttatttccctt 3' |
| mmp2-FP | 5' cgcgtaaagtatgggaacgc 3' |
| mmp2-RP | 5' tctaccgtgtgtgaggccat 3' |
| mmp9-FP | 5' ggacccgaagcggacattg 3' |
| mmp9-RP | 5' cgtcgtcgaaatgggcatct 3' |
| vegfα-FP: | 5' actggaccctggctttactg 3' |
| vegfα-RP: | 5' gcttcgctggtagacatcca 3' |
| hif1α-FP | 5' accttcatcggaaactccaaag 3' |
| hif1α-RP | 5' actgttaggctcaggtgaact 3' |
| gapdh-FP | 5' ccttccgtgttcctaccc 3' |
| gapdh-RP | 5' aagtcgcaggagacaacc 3' |

FP indicates forward primer; RP indicates reverse primer
